# Ligand-controlled growth and stabilization of doped ZnO_2_ nanoparticles for dual antibacterial and enzyme inhibition

**DOI:** 10.1038/s41598-025-15917-6

**Published:** 2025-08-16

**Authors:** Imran Ullah, Reinhard B. Neder, Huma Parwaz, Zul Kamal, Cai-Hong Zhan, Komal Qazi, Inam ud Din, Hari Pokhrel

**Affiliations:** 1 Institute of Condensed Matter Physics, Chair of Crystallography and Structural Physics, Friedrich-Alexander University, Staudt Street 3, 91058 Erlangen, Germany; 2 Department of Physics, University of Science and Technology Bannu, Bannu, KPK Pakistan; 3 Chongqing University of Chinese Medicines, Puguobao Road, 402760 Chongqing, People’s Republic of China; 4 Department of Pharmacy, Shaheed Benazir Bhutto University, Sheringal, 18300 Dir Upper, Khyber Pakhtunkhwa Pakistan; 5 Key Laboratory of the Ministry of Education for Advanced Catalysis Material, College of Chemistry and Materials Science, Zhejiang Normal University, Jinhua, 321004 China; 6 Department of Biotechnology, University of Science and Technology Bannu, Bannu, KPK Pakistan; 7 Department of Physics, University of Peshawar, Peshawar, Khyber Pakhtunkhwa Pakistan; 8 Department of Physics, Friedrich-Alexander University, Erlangen, Germany

**Keywords:** Zinc peroxide nanoparticles, Nucleation, Anti-microbial activity, Acetylcholinesterase enzyme inhibition, Molecular docking, Density functional theory (DFT), Nanoscale biophysics, Nanoscience and technology

## Abstract

Maintaining structural stability in multifunctional nanoparticles (NPs) remain a challenge in nanomedicine. To address this limitation, organic ligand-capped pristine and doped zinc peroxide (ZnO_2_) NPs were synthesized via co-precipitation method for enhanced antibacterial efficacy against Gram-positive bacteria (methicillin-resistant *Staphylococcus aureus* (MRSA) and *Bacillus cereus* (BC)), and inhibition of the acetylcholinesterase enzyme (AChE). The synthesized samples were characterized using complementary characterization techniques. *In-situ* studies confirmed citrate (cit) molecules slow down the nucleation kinetics, while manganese (Mn) and cobalt (Co) doping reduces the optical bandgap from 3.07 eV to 2.89 eV, and 2.79 eV, respectively. Critically, ligand engineering and doping substantially improved bioactivity. ZnO_2_ NPs exhibited dose-dependent antimicrobial activities, with 7.7 ± 0.9 mm and 8.6 ± 0.9 mm zones of inhibition (ZOIs) at 1000 µg/ml concentration against MRSA and BC, respectively. Incorporation of 3% Mn into the ZnO_2_ lattice improved the ZOIs to 8.9 ± 1.7 mm against MRSA and 11 ± 1.9 mm against BC at 1000 µg/ml concentration. Notably, 5% Co-doped with cit capping exhibits the ZOIs of 12.5 ± 2.0 mm against MRSA and 6.4 ± 1.5 mm BC at 1000 µg/ml. 3% Mn-doped ZnO_2_ NPs with dmlt as capping agent showed ZOIs of 10.3 ± 1.7 mm against MRSA and 12.3 ± 1.9 mm against BC at 1000 µg/ml concentration. Furthermore, the anti-acetylcholinesterase enzyme (AChE) activities of the synthesized NPs were assessed. At 125 µg/ml concentration, cit-capped ZnO_2_ NPs inhibits 75.5 ± 0.1% of AChE activity. 3% Mn-doped ZnO_2_ NPs show the inhibition of 73.2 ± 0.2% AChE, enhancing to 82 ± 0.3% upon pent capping. In contrast, 3% Co-doped ZnO_2_ NPs and dmlt-capped 5% Co-doped ZnO_2_ NPs exhibit modest AChE inhibition, with values of 62.4 ± 0.3% and 54.5 ± 0.2%, respectively. Molecular docking studies suggested moderate interaction of ZnO_2_ NPs with phenol-soluble modulins alpha2 (PSMα2), strong interaction with Phospholipase C Regulator (PlcR), and moderate interaction with 1EEA (acetylcholinesterase from *Electrophorus electricus* (electric eel)). This study introduced a novel approach utilizing highly stabilized ZnO_2_ NPs as potent antimicrobial agents and acetylcholinesterase inhibitors.

**Supplementary Information:**

The online version contains supplementary material available at 10.1038/s41598-025-15917-6.

## Introduction

Dementia and antibiotic resistance are two most serious healthcare challenges. Alzheimer’s disease (AD), a progressive and irreversible neurodegenerative disorder, remains without appropriate medical management. The degradation of acetylcholine (ACh), a critical neurotransmitter, by the acetylcholinesterase enzyme (AChE) is a natural process that occurs after signal transmission. However, irregularities in ACh levels lead to various neurological disorders, including AD. Currently, over 55 million people worldwide suffer from various kinds of dementia, and this number is projected to rise to 115.4 million by 2050. Individuals suffering from AD experience symptoms such as memory loss and cognitive decline, ultimately leading to death. Notably, 10% of individuals aged 65 are living with AD and this number increases to 33% for individuals of age 85^1,2^. Various approaches and therapies have been explored to treat AD patients, the inhibition of AChE has emerged as promising strategy. AChE hydrolyzed ACh into choline and acetate after transmitting its message to avoid overstimulation of neurons^3,4^.

Simultaneously, the excessive misuse of antibiotics in the treatment of bacterial infections has led to the emergence of multi-drug-resistance (MDR) bacterial strains. Other contributing factors to antibiotic resistance include intrinsic effects within bacterial species, mutations in chromosomal genes, bacterial evolution, and adaptive behaviors in response to environmental changes. Genetic mutations in genes during drug metabolism often enhance bacterial resilience. Horizontal gene transfer, where resistant bacteria transfer the genes to another bacterial species, is responsible for widespread antibiotic resistance^5–8^. These challenges have spurred efforts to develop efficient AChE inhibitors and novel antimicrobial agents.

Nanotechnology, which emerged in 1959, has gained significant attention in the biomedical field. The high surface-to-volume ratio at the nano-scale makes their properties unique and different from their bulk counterparts^9,10^. Among the various synthesis routes (a matter of choice), the co-precipitation method has many advantages over the others approaches, including environment-friendly, cost-effective, non-toxic, and scalability for bulk production. Common characterization techniques such as X-ray diffraction (XRD), scanning electron microscopy (SEM), UV-Vis spectroscopy, and Fourier-transform infrared spectroscopy (FTIR) are widely used to study NPs^11,12^. Controlling NPs growth is crucial to prevent agglomeration and aggregation, which can compromise their performance. Various methods have been explored to overcome the agglomeration and aggregation^13,14^. One way to address the issue is to modify the surface of NPs using various capping agents (ligand molecules). Capping enhances the potentiality of NPs by providing protective layer against accumulation and aggregation, thereby improving their colloidal stability, dispersibility, and surface functionality, particularly in biological environment. Organic molecules—citrate (cit), 1,5-diphenyl-1,3,5-pentanetrione (pent), and dimethyl-L-tartrate (dmlt)—were used as capping agents to enhance the stability of ZnO_2_ NPs. The carboxyl and hydroxyl groups in cit and dmlt strongly bind to the NPs surface. Pent provides slightly weaker interaction due to limited functional groups, but its aromatic and polar groups can aid molecular interactions, including π-π stacking or dipole interactions^15^.

Various metal and metal oxide NPs have been explored for antimicrobial and AChE inhibition applications. Silver (Ag), gold (Au), copper oxide (CuO), manganese oxide (MnO), and zinc oxide (ZnO) NPs, have demonstrated potential antibacterial and neuroprotective properties. ZnO NPs have been more commonly studied, gaining significant interest due to their biocompatibility, tune surface chemistry, and easy to synthesize, making them a promising candidate for antimicrobial and enzyme inhibition applications. Additionally, their broad-spectrum antimicrobial activities (attributed to reactive oxygen species (ROS), zinc ion release (Zn^2+^) and disruption of bacterial membrane), and as AChE inhibitor (Alzheimer’s-related studies) are explored. However, limited studies about ZnO_2_ NPs have been reported, even though they exhibits greater oxidative potential, making them an effective agent for reactive oxygen species (ROS)-mediated antimicrobial activity. ZnO_2_ is semiconductor material with a wide bandgap ranging from 3.3 eV to 4.6 eV and is used in the rubber industry, plastic production, and other usages. However, ZnO_2_ NPs suffer from stability with phase transition from ZnO_2_ to ZnO, particularly at nanoscale. On other hand, ZnO_2_ releases ROS, which play a critical in disrupting bacterial membranes and inhibiting AChE activity. For instance, Ag NPs, reported potent antimicrobial effects but are limited by dose-dependent cytotoxicity and potential long-term bioaccumulation in human cells. Au NPs have high effectivity but are expensive for widespread use. Similarly, most of the metal oxides (titanium dioxide (TiO_2_), CuO, and iron oxide (Fe_2_O_3_)) have excellent properties, however some are difficult to synthesize in bulk quantity and others may suffer from stability^4,15–29^. In contrast, ZnO_2_ NPs present a more compelling alternative, offering broad spectrum functionality coupled with excellent biocompatibility and potent ROS generation.

This work uses the bottom-up approach (co-precipitation method) to synthesize pure, Mn-doped (Mn_x_), and Co-doped (Co_x_) Zn_1−x_O_2_ NPs stabilized with various organic ligand molecules to enhance their stability and functional properties, addressing the current gap in the literature. The synthesized samples were characterized through XRD, UV–Vis, and FT-IR. The influence of ligand molecules on the size and morphology of the crystallites/NPs was investigated. For structural studies, Rietveld-type refinement was performed using the DISCUS Suite and FullProf Suite^30,31^. The growth dynamics of the NPs were evaluated through *in-situ* experiments. The antimicrobial activities of the synthesized samples were investigated against Gram-positive bacteria, including methicillin-resistant *Staphylococcus aureus* (MRSA) and *Bacillus cereus* (BC). Additionally, the anti-AChE activities of ligand-capped pure ZnO_2_, uncapped doped ZnO_2_, and ligand-capped doped ZnO_2_ NPs were explored. To further support these findings, molecular docking studies were performed to insight into the interaction of the NPs with key bacterial targets and AChE.

## Materials and methods

### Computational details

Density functional theory (DFT) calculations were performed using the SIESTA (Spanish Initiative for Electronic Simulations with Thousands of Atoms) code. The crystallographic information file (CIF) of ZnO_2_ was obtained from the Materials Project, an open-source database. The Atomic Simulation Environment (ASE) GUI was used to create a 2 × 2 × 1 layer of ZnO_2_ system. Convergence test for mesh cutoff, k-points grid and lattice scale factor was performed. The supercell was defined by lattice vectors of 9.9147 Å, 9.9147 Å, and 4.9573 Å, with a 90° angle between the axes and an optimized lattice scale factor of 1.002 Å. The supercell contains a total of 48 atoms, comprising Zn and O species. To obtain a balanced resolution of the Brillouin zone, a 6 × 6 × 6 Monkhorst-Pack grid was used to sample the k-point. Full geometry optimization was carried out using the conjugate gradient method with the force tolerance and energy cutoff of 0.005 eV/Å and 300 Ry, respectively. For the exchange-correlation energy, the Perdew–Burke–Ernzerhof (PBE) functional was used in conjunction with the double-zeta polarized (DZP) basis set, and the electronic temperature was set to 50 meV^32–34^.

### Chemicals and synthesis approaches

All chemicals used for the synthesis of ZnO_2_, and Mn_x_, Co_x_-doped Zn_1-x_O_2_ NPs (x = 0.03, 0.05) were of analytical grade. The properties of NPs, such as size, shape and stability, are influenced by various factors, including organic ligand capping. In this study, cit, pent, and dmlt were chosen as capping agents. Details of the chemicals used and synthesized samples are tabulated in Tables S1 and S2. The NPs were synthesized following the approach described by Imran Ullah et al.^15^. Briefly, 0.5926 g of zinc acetate dihydrate and an optimized amounts of organic ligands were dissolved in a base solution of base containing 5 ml ammonium hydroxide (25% in water) (NH_4_OH) and 3 ml tetra-methyl ammonium hydroxide (25% in methanol) (TMAH), followed by magnetic stirring at 350 rpm for 30 min. The metal salt dissolved quickly. To adjust the pH from 14 to 9–10, which is suitable for NPs formation, 14 ml deionized water (DIW) was added, and stirring continued at the same speed for additional 10 min. Subsequently, 8 ml of hydrogen peroxide (H_2_O_2_) (30% in water) was added slowly. The solution turned cloudy after the addition of 1.5 ml H_2_O_2_ and became white upon the complete addition of 8 ml. The white solution was stirred for additional 30 min. To get the final product, the white solution was centrifuged three times at a speed of 4000 rpm, followed by washing two times with DIW and once with acetone. The final product was placed in a desiccator for 10–12 h to dry completely.

For Co-doped samples (x% = 3, 5) doping, a mixture of the cobalt precursor (cobalt acetate tetra hydrate) and zinc salt was added to the base mixture at the initial stage, followed by the same steps as discussed above. Mn, being a highly oxidizing agent, reacts with H_2_O_2_, producing oxygen bubbles and heat. Using a mixture of Mn salt and zinc precursor in the initial stage produces secondary phases. So slightly different approach was used for Mn-doped samples. First, a pure sample of ZnO_2_ NPs with and without capping agents was prepared. The sample was dissolved in 40 ml of ethanol, and a separate solution of the manganese precursor (manganese acetate tetra hydrate) in 10 ml of ethanol, followed by magnetic stirring at room temperature. The manganese solution was then slowly added to the ZnO_2_ solution, and kept stirring for 40 min. The final product was washed and dried as stated earlier, as shown in Fig. S1.

**Fig. 1:**
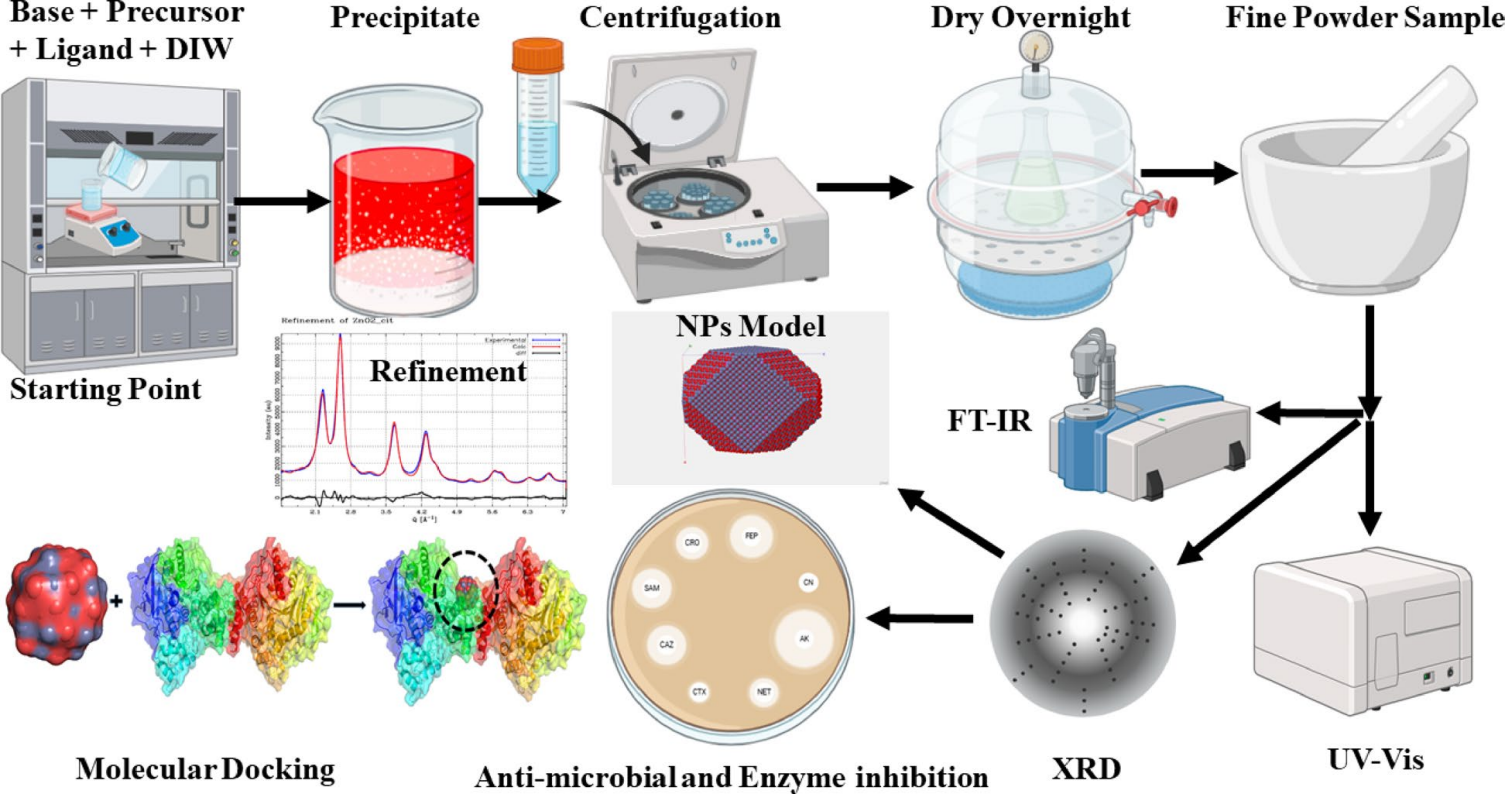
General overview of synthesis, characterization and application of ZnO_2_ NPS.

The 3% and 5% doping levels for Co and Mn represent molar percentage relative to Zn. Precursor masses were calculated by stoichiometric substitution of Zn^2+^ with Co^2+^-/Mn^2+^-. Further details are provided in the Table S3. A general overview of the study is shown in Fig. 1.

The entire reaction was carried out at room temperature. The amount of base mixture of TMAH and NH_4_OH was optimized through a trial-and-error method to achieve stable NPs. The selection of 90 mmol solution containing 0.59265 g of the metal salt was deemed optimal due to the requirement for a robust signal during the *in-situ* study of NPs growth.

### X-Rays diffraction and refinement of synthesized samples

Fine powder of all synthesized samples were measured using two different diffractometers with copper radiations wavelength of K_α1_ = 1.54056 Å and K_α2_ = 1.54439 Å for the first instrument, and K_α1_ = 1.54059 Å and K_α2_ = 1.54441 Å for the second instrument. The X’pert diffractometer was configured with a tube current of 35 mA, a source voltage of 40 kV, a beam opening slit of 10 mm, and incident slits of 1/2, and 1^°^. For the smartlab diffractometer, tube current was set to 160 mA, source voltage to 45 kV, and used soller slits of 2.5^°^. Lanthanum hexaboride (LaB_6_) was used as a standard to determine instrumental parameters.

For structural studies, refinement was carried out using the DISCUS Suite and FullProf Suite. The DISCUS Suite refines models of NPs against experimental XRD data employing *DIFFEV* program, which employ a differential evolutionary algorithm^30,35,36^. The refinement starts by reading the asymmetric unit cell from a CIF, the crystal structure is shown in Fig. S2. The unit cell is then extended along *a*,* b*,* c* directions, and stacking fault are introduced. The surface of the model is decorated with corresponding ligand molecules. The powder pattern of the decorated NPs is calculated using the Debye scattering equation (*DSE*) (1).

1 $$\:I\left(Q\right)=\:\frac{\sum\:_{i}\sum\:_{j}{f}_{i}{f}_{j}\text{sin}\left(Q{r}_{ij}\right)}{Q{r}_{ij}}$$

where I(Q) is the scattering intensity as a function of the scattering vector Q and fi, f_j_ are the atomic form factors of $\:{i}^{th}$, $\:{j}^{th}$ scatterers and $\:{r}_{ij}=$ $\:{r}_{i}-{r}_{j}$.

The Kuplot program, a part of the *DISCUS* package, was used to compare the calculated powder pattern with the observed one. The goodness of fit ($\:{\chi\:}^{2}$) or *R-value* is evaluated for each generation/iteration, and the new set of parameters was transferred internally to *DIFFEV* to proceed with refinement. Further details can be found in^37^. The FullProf Suite was used to optimize crystal structure models by varying atomic positions, Debye-Waller factors, lattice parameters, peak shape parameters, and asymmetry parameters etc. using Rietveld refinement with least squares fitting. It provides Le Bail refinement for profile matching and models diffraction peak morphologies using profile functions such as Pearson VII and Pseudo-Voigt^38^.

### Anti-MRSA and anti-BC activity

Fresh cultures of MRSA were collected from an infection site and initially cultured on Tryptic soya agar (TSA), which was then cultured in Tryptic soya broth (TSB). Fresh cultures were used for all anti-MRSA experiments. The minimum inhibitory concentrations (MIC) were analyzed through the method reported^39^. In this work, MRSA strains were selected for MIC and zone of inhibition (ZOIs) through agar-well diffusion method were measured^40^. MRSA was cultured in a fresh TSB medium for 12 h at 37 °C on a shaker bed (220 rpm). The MIC of ZnO_2_ NPs, 3% Mn-doped ZnO_2_ NPs, 3% Mn-doped ZnO_2_ dmlt NPs, and 5% Co-doped ZnO_2_ cit NPs was determined as per guidelines of clinical and laboratory standards institute (CLSI, 2023) using tetrazolium microplate assay in U-shaped 96-well plates. Bacterial viability kinetics were assessed at different sample concentrations of 1000, 750, 500, 250, 125, 62, 31, 15, 7, 3, 1 µg/ml. Bacterial cultures in the log phase were diluted with fresh medium, and 20 µl of bacteria with optical density (OD) 600 nm of 0.5 McFarland standard (equivalent to 1.5 × 10^6^ CFU/mL) were added to each well of the 96-well plate. TSB with MRSA served as positive control, while samples were added separately to the wells. The plates were incubated overnight, and bacterial growth was monitored at OD 600 nm using a multi-functional microplate reader. The groups treated with DMSO were used as negative control. All experiments were carried out in triplicates.

The ZOIs is the circular region surrounding the antibiotic site and prevents bacterial colonies from growth and development. It is an effective technique for detecting the susceptibility and responsiveness of bacteria to antibiotics. The sensitivity of ZnO_2_ NPs, 3% Mn-doped ZnO_2_, 3% Mn-doped ZnO_2_ dmlt, and 5% Co-doped ZnO_2_ cit against MRSA were determined using^4,41^. MRSA was cultured on TSA plates and grown in TSB medium to an OD 600 nm of 0.5. Fresh agar plates (30 mL) were filled with 200 µL MRSA suspension and allowed to solidify at room temperature. Next, using a sterile cork borer, generate wells in each of these plates with a diameter of 6 mm. Subsequently, add 1000, 750, 500, and 250 µg/mL concentrations of samples ZnO_2_ NPs, 3% Mn-doped ZnO_2_, 3% Mn-doped ZnO_2_ dmlt, and 5% Co-doped ZnO_2_ cit NPs in a clockwise manner. The last well was used as a negative control using a sterile syringe, and the mixture was left undisturbed for proper diffusion at room temperature. The plates were incubated in an incubator for 18–24 h at 37 °C and the diameter of inhibition zones were measured in millimeter (mm).

*Bacillus cereus* bacteria were obtained from Microbiology and Immunology core Laboratory, Khalifa University, Abu Dhabi, United Arab Emirates and preserved at 4 °C. For the experiments, fresh cultures were grown in nutrient broth for 24 h at 37 °C and stored at 4 °C for preservation. For the antimicrobial activities, nutrient agar was used.

### Methodology for ache inhibition

AChE inhibition was assessed using approach described by Ullah et al.^4^ for ZnO NPs. Acetylthiocholine, a structural analog of acetylcholine, was used as the substrate for the AChE enzyme. The enzyme-substrate reaction began immediately upon substrate addition, and hydrolysis was monitored by the formation of a yellow color. Hydrolysis of acetylthiocholine by AChE results in thiocholine (containing a sulfur atom bonded to hydrogen atom (SH group)) and acetate. Ellman’s reagent reacts with the thiol group, releasing 5-thio-2-nitrobenzoic acid (TNB), a yellow colour compound. Hydrolysis rates (*V*) were measured at 1 mM acetylthiocholine (*S*) concentration in 1 ml assay mixture with 50 mM phosphate buffer at pH 7.4, 10 mM *DTNB*, and various concentrations (75, 100, and 125 µg/ml) of ZnO_2_ cit, ZnO_2_ pent, ZnO_2_ dmlt, and Mn_x_-, Co_x_- doped Zn_1 − x_O_2_ (for x = 0.03, x = 0.05) with cit-, pent-, dmlt- apping were incubated at 25 °C for 5 min. The absorbance was measured at wavelength 421 nm.

### Molecular docking

To validate the antimicrobial and anti-AChE activities of ZnO_2_ NPs, molecular docking was performed to understand the interactions between the NPs and the targeted protein/enzyme, phenol-soluble modulins alpha2 (PSMα2) for MRSA, phospholipase C regulator (PlcR) for BC, and 1EEA (acetylcholinesterase from *Electrophorus electricus* (electric eel)). The receptors structures were obtained from the RCSB protein data bank. Further details are provided in the supplementary materials.

### Statistical analysis

All assays were run in triplicate (*n* = 3), with results shown as the mean ± standard error (SE). To compare groups, a one-way analysis of variance (ANOVA) test was conducted. A p-value of < 0.05 was considered significant, where * indicates *p* < 0.05, ** indicates *p* < 0.01, *** indicates *p* < 0.001, **** indicates *p* < 0.0001, and ns represents non-significant.

## Results and discussions

### Structural optimization of ZnO_2_ (221) supercell and surface effects

**Fig. 2:**
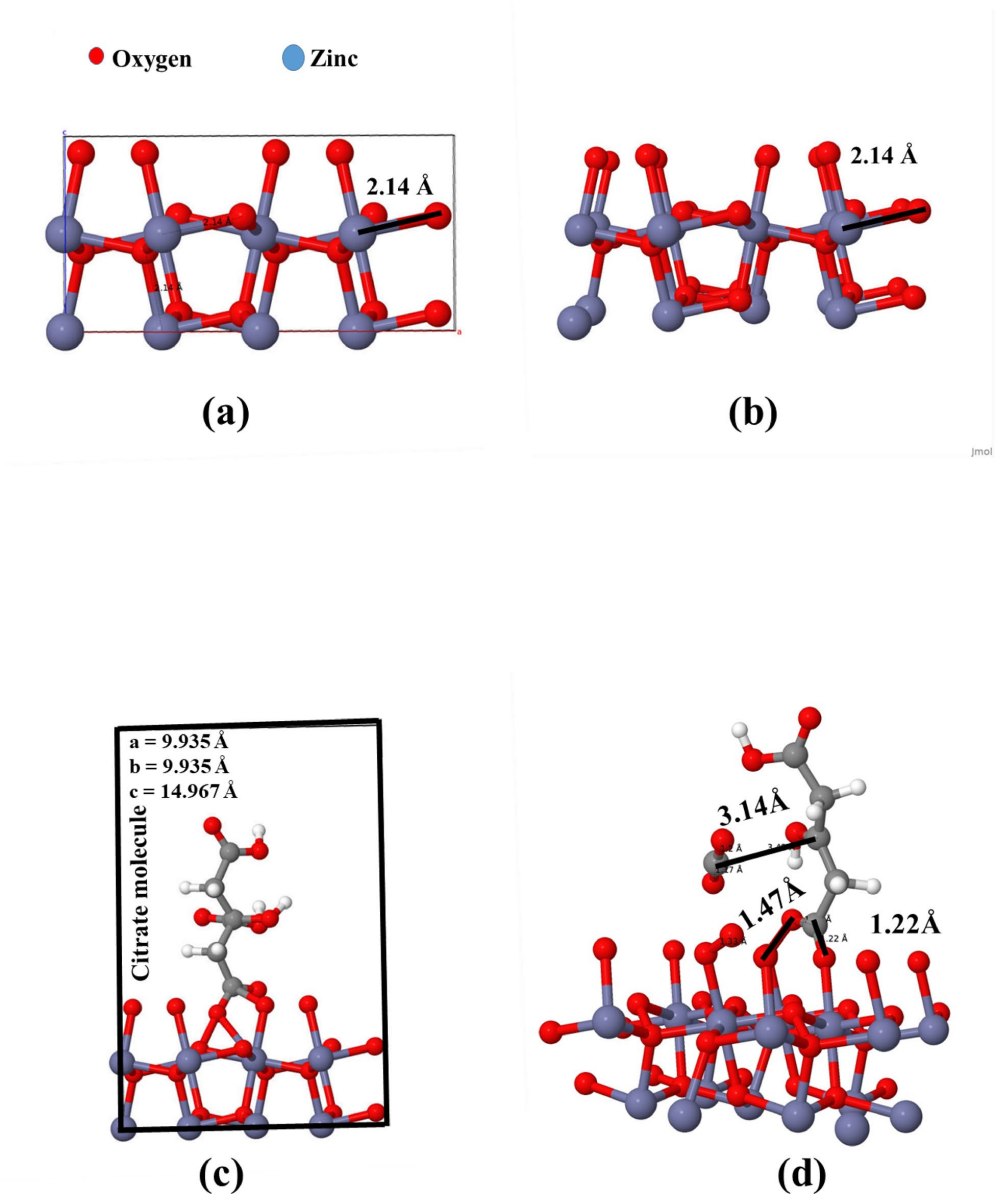

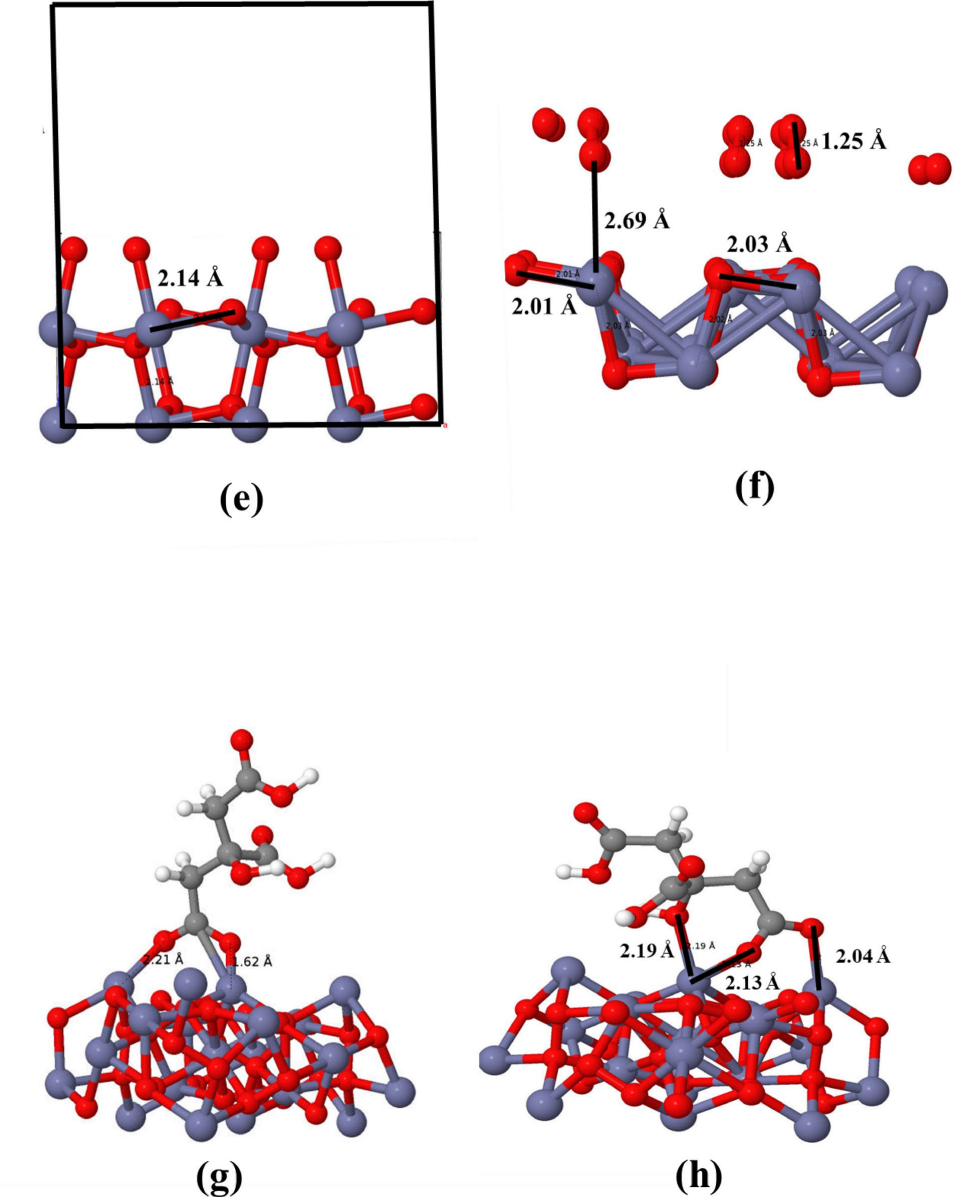
Geometry of ZnO_2_ supercell (2 × 2 × 1): (**a**) before optimization and (**b**) after optimization, showing no significant changes in bond lengths. Cit molecule adsorption on ZnO_2_ (oxygen terminated) (221) surface before (**c**) and after optimization (**d**) illustrating CO_2_ dissociation and unintended interactions between oxygen atoms of cit and ZnO_2_ surface. (**e**,**f**) ZnO_2_ (221) surface relaxation with 10 Å vacuum, highlighting spontaneous surface oxygen movement and Zn–O_2_ bond formation (2.69 Å) after free atomic relaxation. (**g**,**h**) Cit adsorption on ZnO_2_ (111) surface, showing successful Zn–O coordination bonds (2.04–2.19 Å) and hydrogen bonding due to cit molecular bending.

The adsorption behavior of the cit molecule on the ZnO_2_ surface was investigated through DFT calculations implemented via the SIESTA code. A ZnO_2_ supercell (2 × 2 × 1) was constructed from the optimized unit cell and relaxed. The optimized structure was analyzed before any further modification to confirm structural stability. After optimization, no significant changes were observed in the bond lengths (Zn–O = 2.14 Å), as shown in Fig. 2 a,b. The results indicated that the ZnO_2_ (221) structure maintained a bulk-like character under periodic boundary conditions, implying that the supercell behaves more like an extended bulk structure rather than an exposed surface. To make the observation more realistic, a vacuum of 10 Å was added along the c-axis to eliminate interactions between periodic images. A cit molecule was placed at the middle of the simulated box on the top of ZnO_2_ surface in an initial configuration. The supercell atoms were fixed, while cit molecule was allowed to move and find their position for optimal adsorption. After interaction, it was observed that CO_2_ molecules moved away from the cit molecule (decarboxylation reaction). Results in undesired interactions between the O atoms on the ZnO_2_ surface and the carboxylic acid groups of the cit molecule, as shown in Fig. 2 c,d. In conclusion, the ZnO_2_ supercell (221) with a 10 Å vacuum was allowed to optimize first as there was a possibility that the adsorption energy and bonding mechanism did not fully reflect real surface interactions. Figure 2e,f showed the geometry before and after optimization, where all atoms were allowed to move freely, enabling the system to reach a more stable. The results indicated notable atomic rearrangements with surface oxygen atoms moving upwards, indicating a spontaneous relaxation mechanism. The bond length of 2.69 Å was observed between Zn atoms on the surface and the O_2_ molecule with a slight reduction in Zn–O in the supercell of ZnO_2_. Such an upward shift of O atoms indicated that ZnO_2_ surfaces are highly dynamic, which can significantly influence their properties. For the mixed (Zn and O) terminated surface, the ZnO_2_ supercell was cut through the (111) plane, leading to diverse adsorption sites. The system was optimized with a 10 Å vacuum normal to the (111) plane, resulting in significant atomic rearrangement. A pre-optimized cit molecule was placed above the surface, with the ZnO_2_ supercell atoms constrained while the cit molecule was allowed to move freely. The cit molecule successfully anchored onto the ZnO_2_ (111) plane by forming several Zn–O bonds with bond lengths ranging from 2.04 to 2.19 Å. Additionally, bending of cit molecule was observed, leading to the formation of hydrogen bonds, as shown in Fig. 2g,h. Ullah et al.^28^ found the bidentate attachment of the cit molecule to the ZnO surface through the carboxylic acid group, with two different Zn–O bond lengths of 2.80 Å and 2.19 Å.

### XRD of ZnO_2_ and doped ZnO_2_ NPs with different ligands molecules

**Fig. 3:**
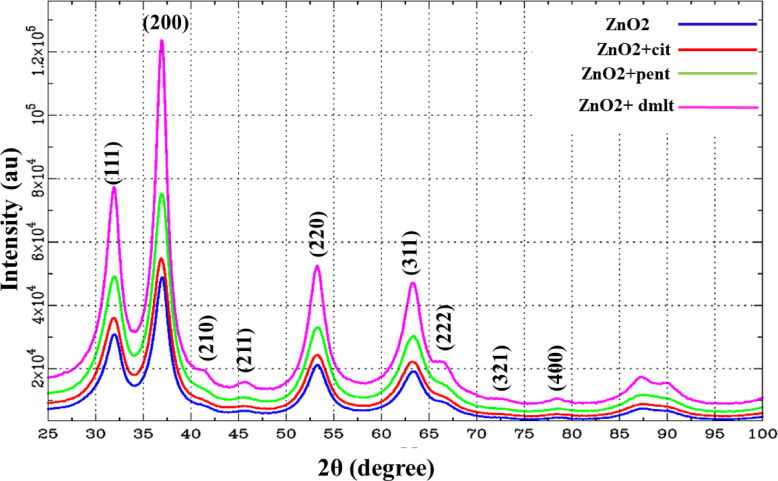
XRD of pure ZnO_2_ NPs with and without organic ligand capping.

X-ray diffraction XRD of the synthesized samples were performed to confirm single phase crystallization, NPs formation, and to gain insights into structural analysis, including size, shape, and the effect of ligand molecules on the size of crystallites/NPs. Instrumental parameters were determined by measuring lanthanum hexaboride (LaB_6_). The XRD pattern for the pure phase is shown in Fig. 3. Peaks observed at 2θ ≈ 31.87°, 36.96°, 41.52°, 45.69°, 53.27°, 63.43°, 66.61°, and 72.75° correspond to reflections from the (111), (200), (210), (211), (220), (311), (222), and (321) planes, respectively. No additional peaks belonging to impurities or other phases were observed. The high intensities and broad full width at half maximum (FWHM) of the observed peaks indicated the pronounced crystallinity and formation of crystallite/NPs, respectively. The impact of organic ligand molecules on the size of crystallites/NPs was reported as per the literature of Imran Ullah et al.^27^. The results demonstrated the usage of cit as capping agent produced the smallest NPs. Furthermore, XRD patterns for Mn-doped and Co-doped ZnO_2_ NPs are depicted in Fig. S3a–d, signified single phase crystallization for all doped samples with a slight peak shifting to higher 2θ values upon Mn doping. Mn can exist in an oxidation state ranging from + 2 to + 7, leading to varying ionic radii. The peak shift to higher 2θ values suggested lattice contraction, which may be likely due to the specific oxidation state of Mn within the ZnO_2_ lattice. Complex behaviour was observed upon Co-doping. The peaks shifted to higher 2θ values at lower dopant concentration, and to lower 2θ values at higher dopant concentration. At lower concentrations, the contraction of the lattice occurred due to the distortion and strain induced by Co ions incorporation, while peaks shifted to lower 2θ values can be justified by the larger ionic radii of Co ions. The corresponding peak positions are provided in Tables S4, and S5. Moreover, higher dopant concentration lead to formation of larger size crystallites/NPs with some surface strain (upon 5%Co-doping), contributing to *FWHM* broadening. Such structural change may improve the functional properties of ZnO_2_ crystallites/NPs.

### Refinement

**Fig. 4:**
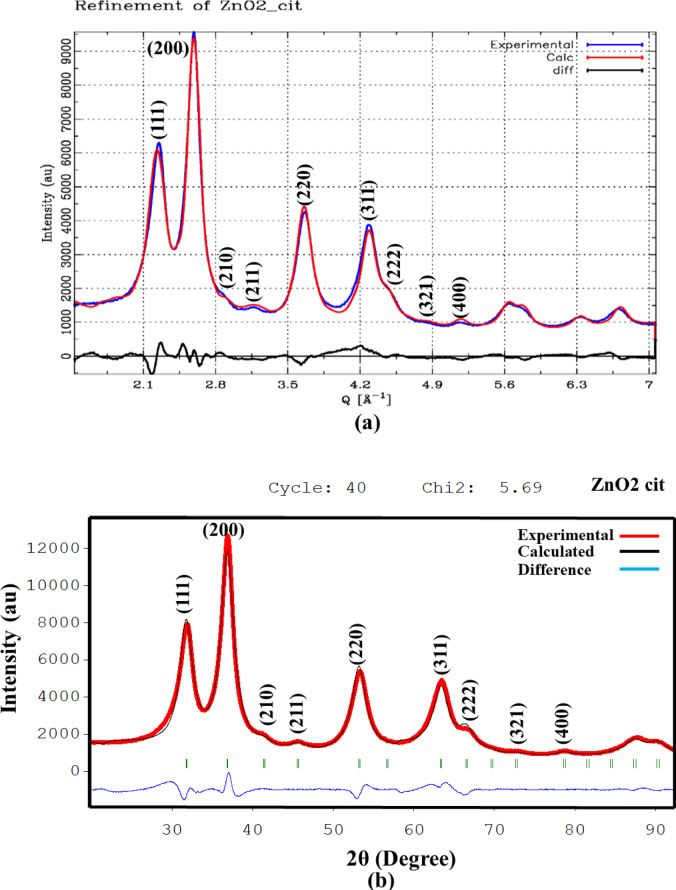
XRD data collected via two different diffractometers and the corresponding refinement via DISCUS Suite (**a**) FullProf Suite (**b**).

XRD data refinement was performed using the DISCUS Suite (Fig. 4a) and FullProf Suite (Fig. 4b). Lattice parameter *a* (*P_lata*), oxygen position (P_x_o), Debye-Waller factor (P_biso), diameter along *a* (P_aa_dia), *b* (P_bb_dia) and *c* direction (P_cc_dia) were our targeted parameters to refine via the DISCUS Suite. The model for crystallites/NPs was simulated 40 times, decorating it with the corresponding ligand molecules. The calculated powder patterns were averaged and compared with the experimental data for each generation/iteration. A minimum and maximum range, along with a starting and stopping value, was provided for every parameter. For doped samples, an optimal amount of Zn atoms was replaced with the dopant atoms before calculating the corresponding powder pattern. The refinement process began with a good initial guess, and the goodness of fit was determined after each generation/iteration until convergence or the predefined number of generations was reached. The refined parameters are provided in Fig. S4 and Table S6. The calculated lattice parameter *a* was 4.87 Å for all except ZnO_2_ NPs capped with cit molecules. Utilizing cit as capping agent, resulting in formation of smallest NPs with induced strain due to size effects, leading to shrinking the lattice and altering d-spacing between crystal planes, as described by the following equation:

$$\:d=\frac{a}{\sqrt{{h}^{2}+{k}^{2}+{l}^{2}}}$$

where a is lattice constant, d is the inter planer (*hkl)* spacing.

**Fig. 5:**
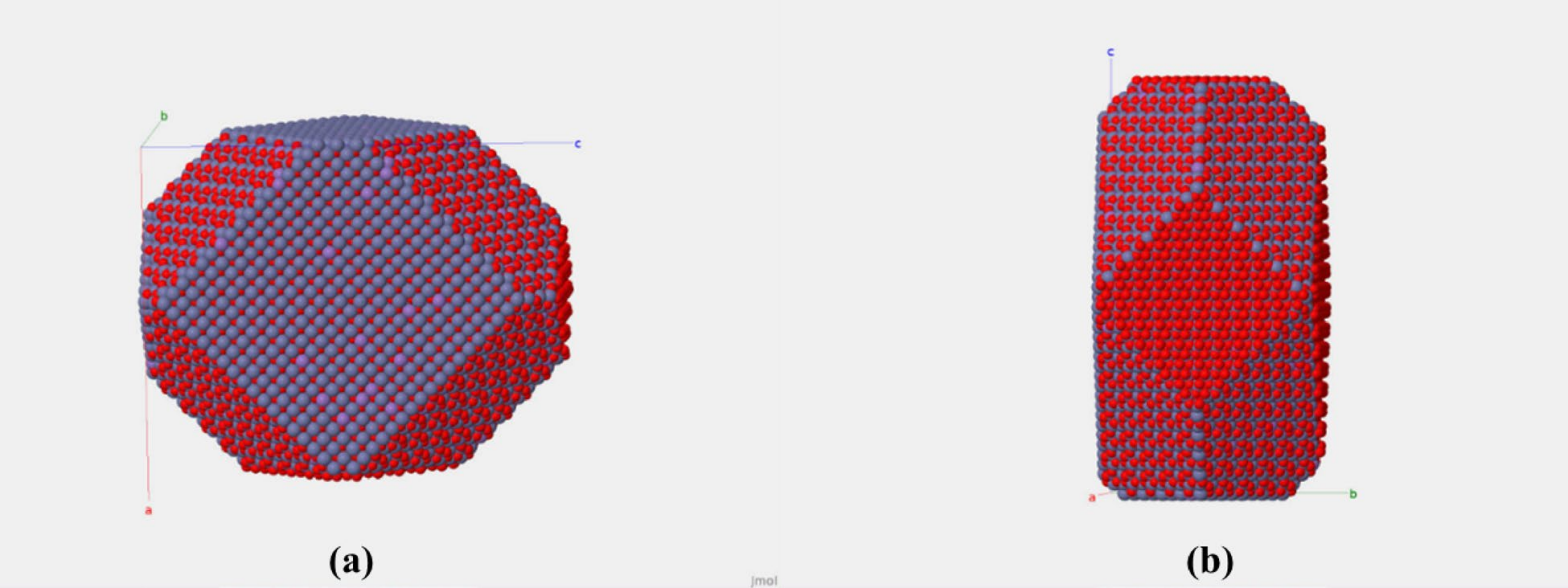
ZnO_2_ NPs model obtained from XRD data refinement, showing the best fit to the experimental XRD pattern. (**a**) Cuboctahedron shape (**b**) Polar and non polar surfaces.

**Fig. 6:**
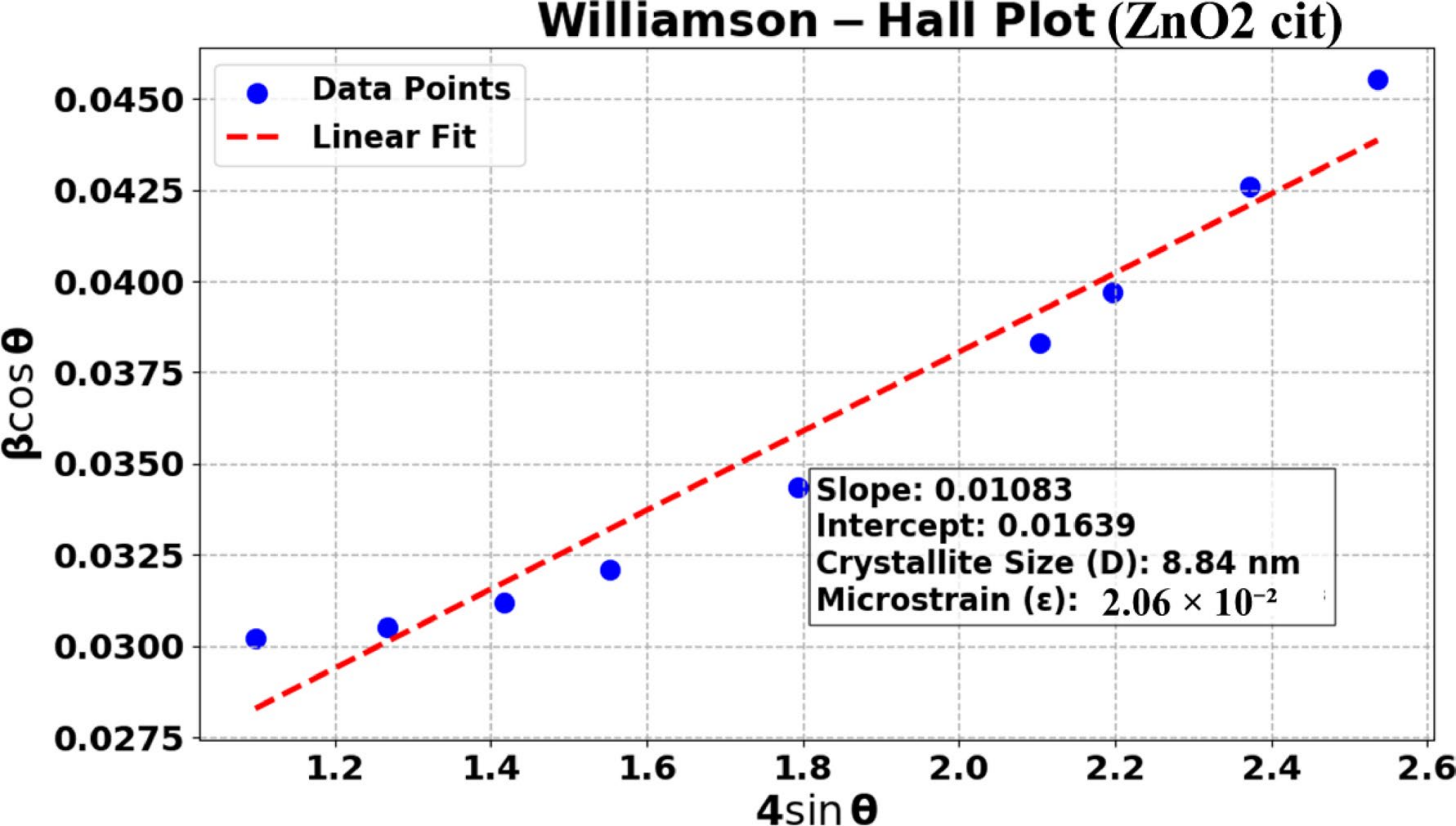
Plot of β_hkl_cosθ versus 4Sinθ plot for cit-capped ZnO_2_ NPs.

The refined diameters are < 10 nm for pure phase. A slight variation in the lattice parameter after Mn, Co-doping was observed with an increase in the size of crystallites/NPs. The largest refined diameter for Mn-doped and Co-doped samples were 11.30 nm and 12.20 nm, respectively. The final model that best fit to the experimental data (Fig. 4a) is depicted in Fig. 5a,b. The refinement suggested the formation of cuboctahedron crystallites/NPs. Additionally, the crystallites/NPs size and microstrain were calculated using Williamson–Hall (W–H) (Fig. 6) plot using the following relation:

$$\:{\beta\:}_{hkl}Cos\theta\:=\frac{k\lambda\:}{D}+4\epsilon\:Sin\theta\:$$

where β_hkl_ is the FWHM of reflection hkl, k (0.94) is shape factor, λ is the wavelength of incident X-rays, D is the crystallite/NPs size (average), ε is the microstrain.

$$\:D=\:\frac{k\lambda\:}{{Y}_{intercept}}$$

$$\varepsilon = \frac{\beta\:}{4tan\theta\:}$$

The calculated crystallite size was 8.84 nm, with a microstrain of 2.06 × 10^− 2^. These results are in close agreement with the Rietveld type of refinement using DISCUS Suite.

### UV-Vis spectroscopy

**Fig. 7:**
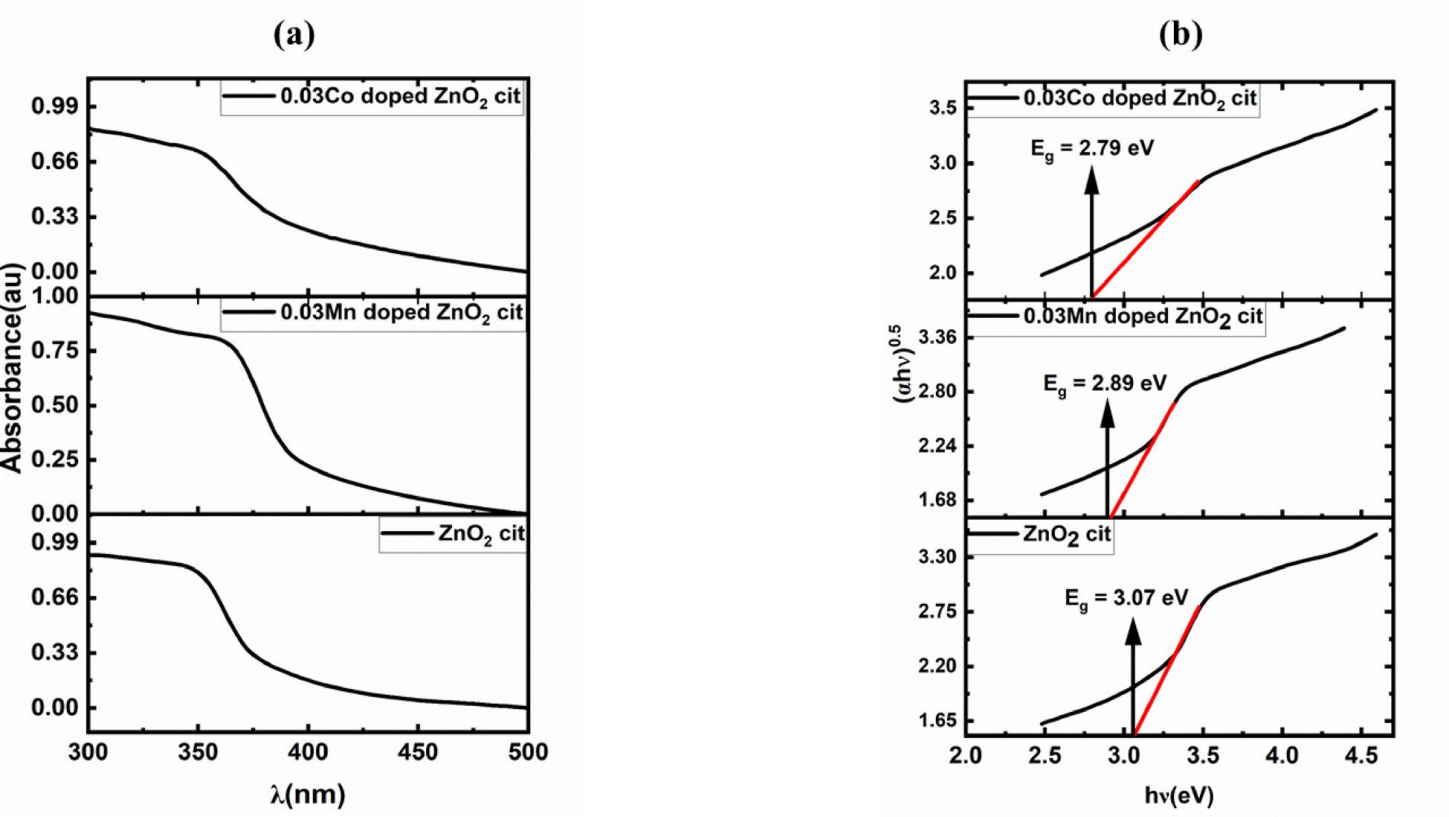
UV–Vis spectrum (**a**) and Tauc’s relation (**b**) of ZnO_2_ cit and Mn, Co-doped ZnO_2_ NPs.

The UV–Vis spectroscopy of cit-capped ZnO_2_ and 3% doped (Mn and Co) ZnO_2_ NPs are depicted in Fig. 7. For the pure phase (ZnO_2_ with cit as ligand molecules), a broad absorption peak was observed in the range of 347 nm to 349 nm. A red shift in the absorbance peak (broad) was observed upon Mn (358 nm) and Co doping (353 nm). The red shift supported the incorporation of dopant into the ZnO_2_ lattice and can be attributed to the Burstein-Moss effect. Dopants introduced additional states near the Fermi level, partially contributed to the valance band and primarily to the conduction band. These additional states from dopant reduced the optical bandgap^42^. The broadening of the absorption peak may be due to the size of the crystallites/NPs or the size distribution. It has been observed that the 3% doping in both cases (Mn and Co-doping) produced tiny particles compared to the pure phase. To find the estimated optical bandgap energy (E_g_), Tauc’s relation was plotted, as shown in Fig. 7.

2 $$\:\left(\alpha\:h\nu\:\right)=A.{(h\nu\:-{E}_{g})}^{n}$$

where α is the absorption coefficient. hν is the incident photon energy. E_g_ is the optical bandgap.

The strong hybridization between the d-orbital of Zn and the p-orbital of O made ZnO_2_ an indirect semiconductor with optical bandgap of 3.07 eV, where the upper part of the valance band located at Γ point and the lower part of the conduction band was situated in-between Γ and R, reduced to 2.89 eV and 2.79 eV upon Mn and Co-incorporation. The results are in close agreement with our previous findings^15^.

### Growth kinetics of ZnO_2_ NPs and cit-capped ZnO_2_ NPs

**Fig. 8:**
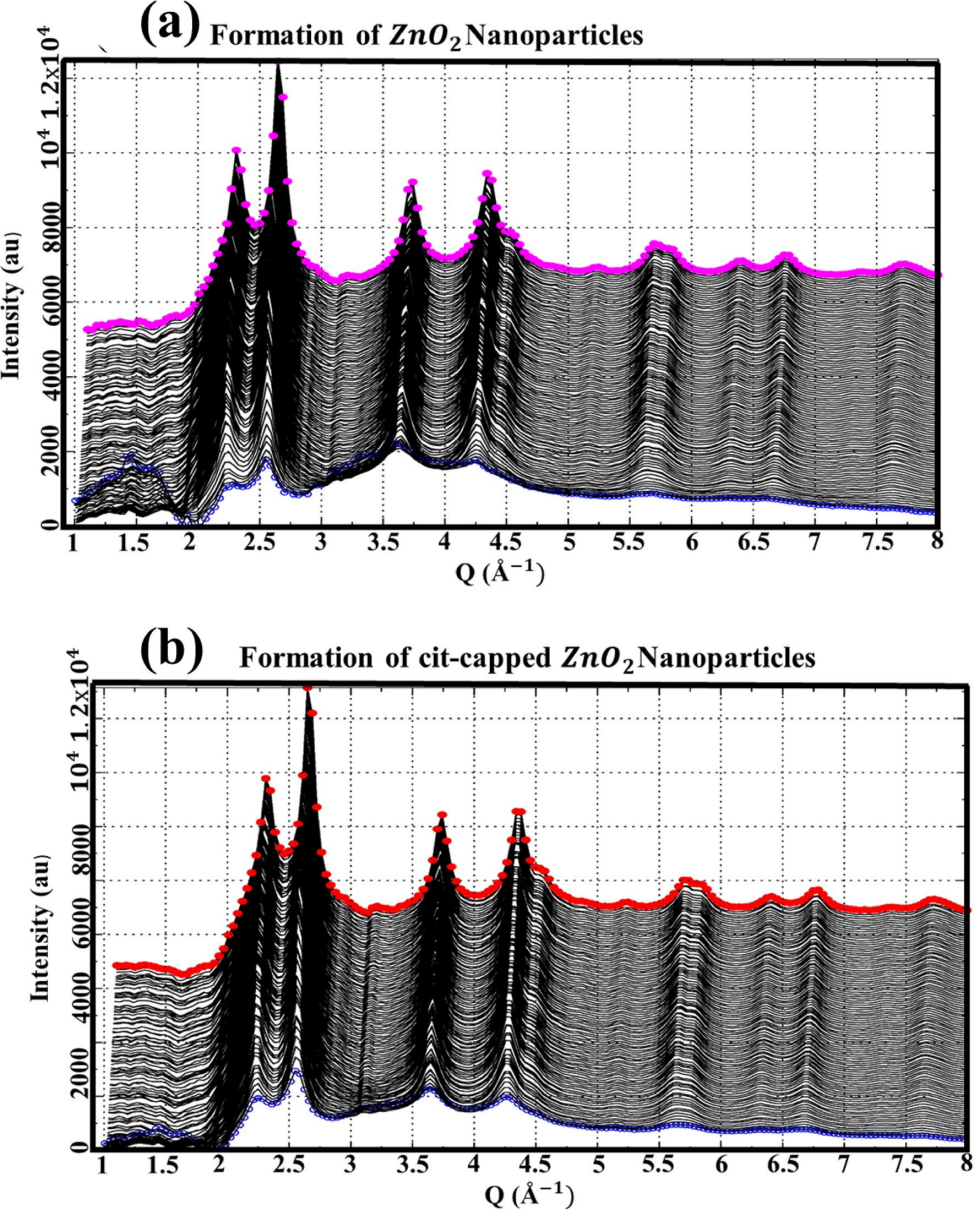
XRD patterns depicting the formation of ZnO_2_ NPs (**a**) and cit-capped ZnO_2_ NPs (**b**). The evolution of structural features with reaction time highlights the crystalline growth, phase stabilization, and surface functionalization due to cit-capping.

To gain insight into the growth kinetics of ZnO_2_ NPs, an *in-situ* experiment was conducted at the European Synchrotron Radiation Facility (ESRF) in France. Data were collected for the synthesis of ZnO_2_ NPs and background signals, including contributions from signals of the solvent (a mixture of base NH_4_OH and TMAH, DIW, and H_2_O_2_), the Kapton sheet, and air scattering, which were subsequently subtracted for comprehensive analysis. The intensity (au) versus scattering vector (Q (Å^−1^) was plotted for the cleaned data, with a slight offset y-axis. The data collected at different time intervals revealed the structural evolution of the NPs. Figure S5a,b showed the raw data without background subtraction, while Fig. 8a,b presented the clean data, without background contribution, indicating the time-resolved evaluation of ZnO_2_ NPs and cit-capped ZnO_2_ NPs.

**Fig. 9:**
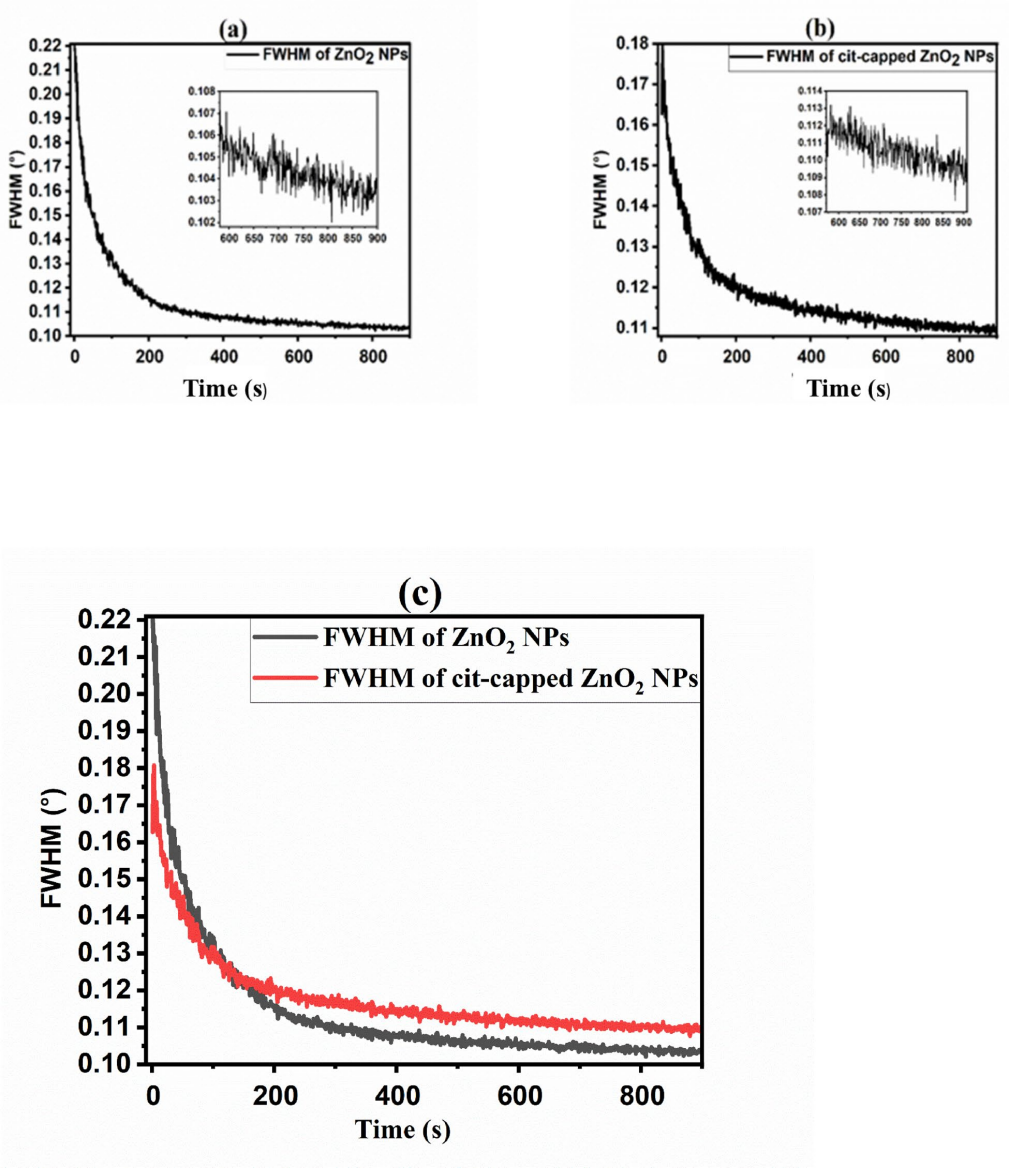
ZnO_2_ NPs growth without ligand molecule (**a**) and with cit-capping (**b**).

Normally, the NPs synthesis included three different evolution periods, namely, nucleation and pre-nucleation, early growth stage, and late growth stage. When the synthesis reaction began, the conversion of the reactants to NPs precursors responsible for the formation of solid nuclei were initiated. As the process progressed, these precursors reached a supersaturation threshold, resulting in spontaneous condensation and the formation of solid nuclei within the liquid medium. The period of nucleus production was contingent upon the reactants employed, such as those for ZnO_2_ NPs and cit-capped ZnO_2_ NPs, resulting in discrepancies in the initial size distribution of the nuclei. In the initial development phase, the nuclei developed into NPs, and phenomena such as Ostwald ripening (where smaller particles dissolve to facilitate the growth of larger ones) and coalescence (the merging of particles) may have transpired due to the elevated surface energy of small NPs. Cit ions are typically carboxylate anions (–COO^−^) and may form coordination bonds with metal ions (Zn^2+^) on the surface of the NPs. Ullah et al.^4,28^ described several attachment scheme for cit-capped ZnO NPs. Among them chelate interaction forming a stable surface complex, which prevented excessive aggregation and growth of the NPs. Moreover, cit molecule consisted of three carboxyl groups. The presence of such molecules on the surface created a physical barrier around them (grows in controlled manner), preventing them to grow rapidly compared to ZnO_2_ NPs without capping agent, where decreased rate of FWHM suggested rapid growth of NPs (FTIR is shown in Fig. S6). This can be attributed to agglomeration (uncontrolled growth), leading to large crystallites/NPs. Cit ligands that associate with ZnO_2_ NPs stabilized smaller particles, leading to a reduced FWHM of their size distribution in contrast to uncapped ZnO_2_ NPs. As NPs increased in size (exceeding 120 s), their surface energy markedly diminished, hence mitigating Ostwald ripening and lessening interparticle clumping. In this advanced growth phase, continued expansion and intra-particle restructuring prevailed. The uncapped ZnO_2_ NPs reflected greater flexibility in reconstruction and growth, ultimately resulting in the formation of nanocrystals. As a result, at this stage the FWHM of uncapped ZnO_2_ NPs was narrower than that of cit-capped ZnO_2_ NPs, as illustrated in the linked Figs. 8a,b and 9a–c. The blue and pink data set in Fig. 8a highlighted the initial and final stages of ZnO_2_ NPs formation without capping agent. Similarly, in Fig. 8b, the blue circle data set represented the starting point and the red circle data set showed the final stage of cit-capped ZnO_2_ NPs. The results were supported through the DFT calculations.

### Antimicrobial activity

The antimicrobial activity of pure ZnO_2_ NPs (without ligand molecules) and 3%, 5% Mn and Co-doped ZnO_2_ NPs (with and without ligand molecules) were evaluated. Ciprofloxacin drug was used as the standard drug. All samples were tested against Gram positive bacteria (MRSA and BC). The thick peptidoglycan layer in the cell walls of bacteria contributed to their resistance. The results showed that ZnO_2_ NPs (without ligand molecules) were active against both strains, exhibiting the zone of inhibitions (ZOIs) of 7.7 ± 0.9 mm against MRSA and 8.6 ± 0.9 mm against BC at concentration of 1000 µg/ml. The incorporation of Mn into the ZnO_2_ lattice enhanced the activity against both strains, resulting ZOIs of 8.9 ± 1.7 mm against MRSA and 11 ± 1.9 mm against BC at 1000 µg/ml. Cit-capped, 5% Co-doped ZnO_2_ NPs reported ZOIs of 12.5 ± 2.0 mm and 6.4 ± 1.5 mm at 1000 µg/ml against MRSA and BC respectively. In the case of dmlt-capped 3% Mn-doped ZnO_2_ NPs, the maximum ZOIs 10.3 ± 1.7 mm was observed at 1000 µg/ml against MRSA and 12.3 ± 1.9 mm against BC at 1000 µg/ml. The standard drug (ciprofloxacin) was used as the positive control at the same concentrations as the tested NPs and demonstrated pronounced inhibition against both strains. All samples reported dose dependent inhibitory activities. The ZOIs (in mm) are depicted in Figs. S7–14 and tabulated in Table 1. This improved activities aligns with earlier studies on Co-doped ZnO NPs, showing enhanced antimicrobial performance attribute to increased ROS generation and optimized surface interactions^43,44^. However limited studies have been reported on ZnO_2_ NPs, particularly on doped ZnO_2_ NPs.

**Table 1:** Antimicrobial activities of ZnO_2_ NPs and doped ZnO_2_ NPs (with and without ligand capping) against MRSA (a) and BC (b). All experiments were performed in triplicates (N = 3), while the concentrations were presented as (μg/ml)

Compounds	Zone of inhibition (mm) for Methicillin-resistant *Staphylococcus aureus*, concentration = µg/ml, *N* = 3 (triplicates)			
	250 µg/ml	500 µg/ml	750 µg/ml	1000 µg/ml
(a)				
ZnO_2_-NPs	6.1 ± 1.5	6.2 ± 1.7	7.2 ± 1.6	7.7 ± 0.9 (MIC)
3% Mn-doped ZnO_2_	6.2 ± 2.4 (MIC)	6.3 ± 2.1 (MBC)	7.6 ± 2.1	8.9 ± 1.7
5% Co-doped ZnO_2_ cit	9.2 ± 1.7	9.5 ± 1.5 (MIC/MBC)	12.2 ± 2.7	12.5 ± 2.0
3% Mn-doped ZnO_2_ dmlt	6.2 ± 2.6 (MIC/MBC)	6.5 ± 2.0	8.3 ± 2.6	10.3 ± 1.7
Negative control	0.0 ± 0	0.0 ± 0	0.0 ± 0	0.0 ± 0
Ciprofloxacin	13.3 ± 2.0	13.3 ± 2.6	15.4 ± 2.4	14.2 ± 3.0

Compounds	Zone of inhibition (mm) for *Bacillus cereus* (BC), concentration = µg/ml, *N* = 3 (triplicates)			
	250 µg/ml	500 µg/ml	750 µg/ml	1000 µg/ml
(b)				
ZnO_2_-NPs	7.5 ± 2.0	7.9 ± 1.7	8.0 ± 2.1	8.6 ± 0.9 (MIC)
3% Mn-doped ZnO_2_	9.0 ± 1.6	10 ± 2.1 (MIC)	10 ± 2.6	11 ± 1.9
5% Co-doped ZnO_2_ cit	6.2 ± 1.7	6.3 ± 2.1 (MIC/MBC)	6.3 ± 1.5	6.4 ± 1.5
3% Mn-doped ZnO_2_ dmlt	6.2 ± 2.4 (MIC)	6.3 ± 2.2 (MBC)	11.4 ± 2.3	12.3 ± 1.9
Negative control	0.0 ± 0	0.0 ± 0	0.0 ± 0	0.0 ± 0
Ciprofloxacin	14.1 ± 2.5	15.5 ± 2.6	15.6 ± 2.6	17.5 ± 1.9

**Fig. 10:**
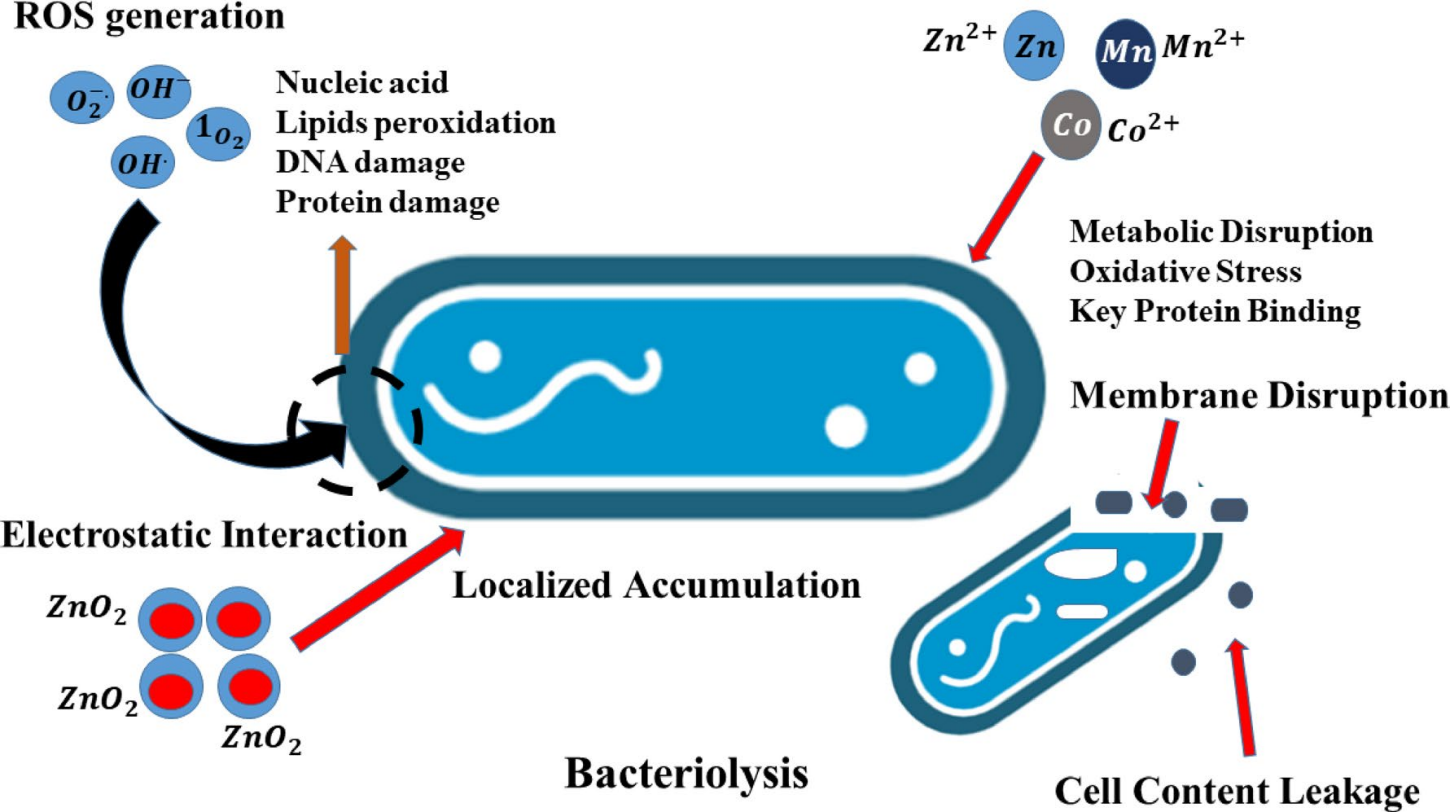
The bacteriolysis mechanism of ZnO_2_ and doped (Mn–/Co–) ZnO_2_ NPs.

The exact mechanism of bacteriolysis using NPs remains a fruitful debate. Few approaches have been proposed, with the most common being the generation of reactive oxygen species (ROS) which inhibited the bacterial growth. The possible ROS included superoxide radicals ($\:{\text{O}}_{2}^{-\cdot\:}$), hydroxyl ions ($\:{\text{O}\text{H}}^{-}$), hydroxyl radical ($\:{\text{O}\text{H}}^{\cdot\:}$), and singlet oxygen (^1^$\:{\text{O}}_{2}$). Among these, $\:{\text{O}\text{H}}^{\cdot\:}$ is the most reactive. Two hydroxyl radicals can recombine to form H_2_O_2_, a highly oxidizing agent. ROS can damage organic molecules, including nucleic acid, lipids, and DNA. Metal ions, Zn^2+^, Mn^2+^and Co^2+^, also contribute to the toxicity of Mn-, Co-doped ZnO_2_ NPs towards bacteria. Metal ions can easily penetrate through negatively charged bacterial cell membranes and interact with various functional groups, disrupting cellular metabolism. Doping with transition metals improve bacterial inhibition, as their toxicity to bacteria is well-documented^20,45–47^. These ions can induce oxidative stress, which initially slows down bacterial growth and eventually leads to cell death. The penetration of metal ions not only results in disrupting the membrane but can also clump the contents of the cell together. Another proposed mechanism is the electrostatic attraction between positively charged ZnO_2_ NPs and the negatively charged bacterial membranes, leading to bacteriolysis. Studies have shown that bacterial cell walls contain anionic surface domains instead of continuous layers, resulting in localized regions with a higher concentration of NPs. This leads to structural damage to the cell wall, further contributing to bacterial cell death^48^. The bacteriolysis mechanism is shown in Fig. 10.

**Fig. 11:**
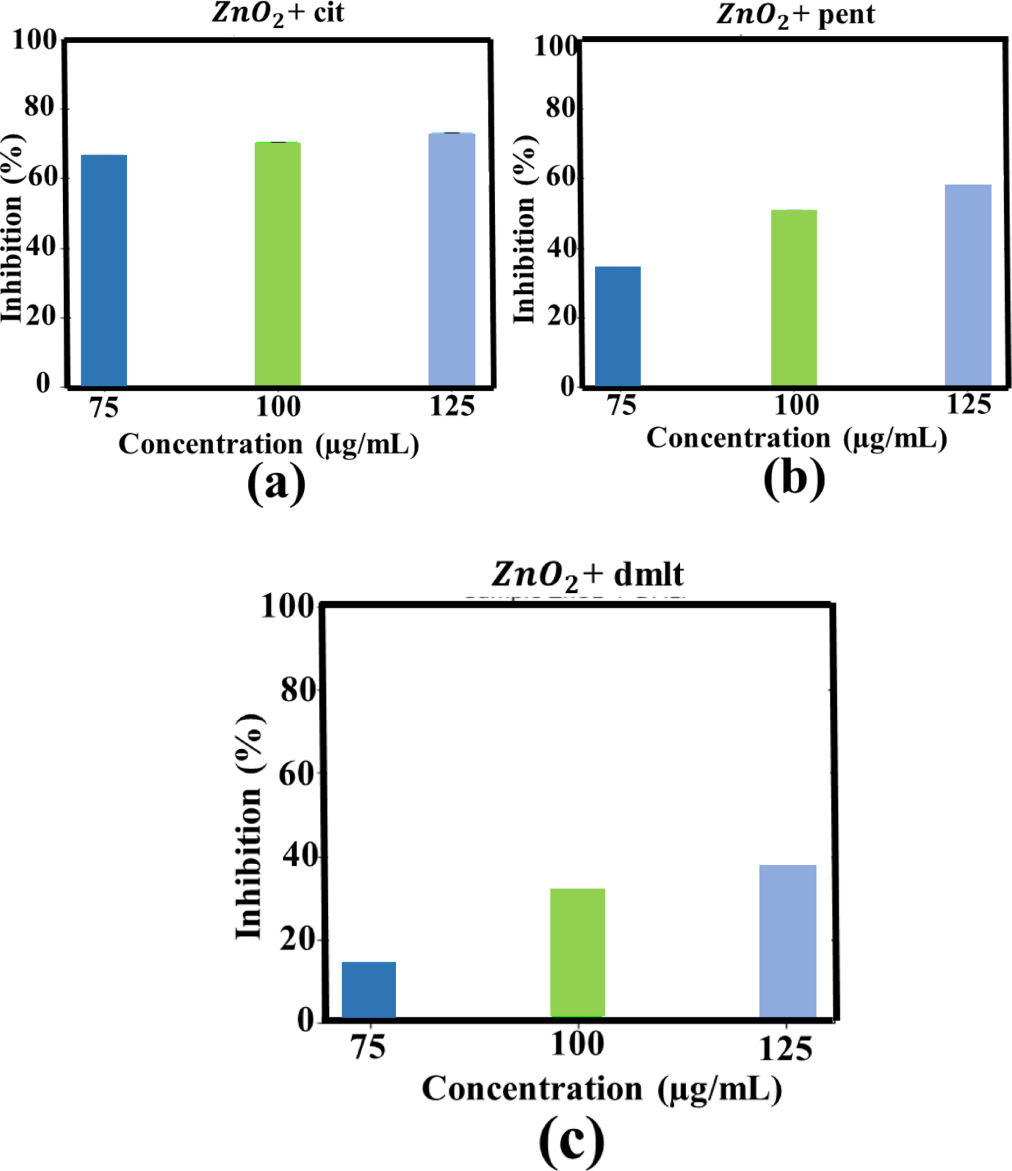
% inhibition of AChE enzyme of cit-capped ZnO_2_ NPs (**a**), pent-capped ZnO_2_ NPs (**b**), and dmlt-capped ZnO_2_ NPs (**c**). All results are presented as mean ± SE (*n* = 3). Statistical significance was evaluated using one-way ANOVA followed by Tukey’s post hoc test (*p* < 0.05).

**Fig. 12:**
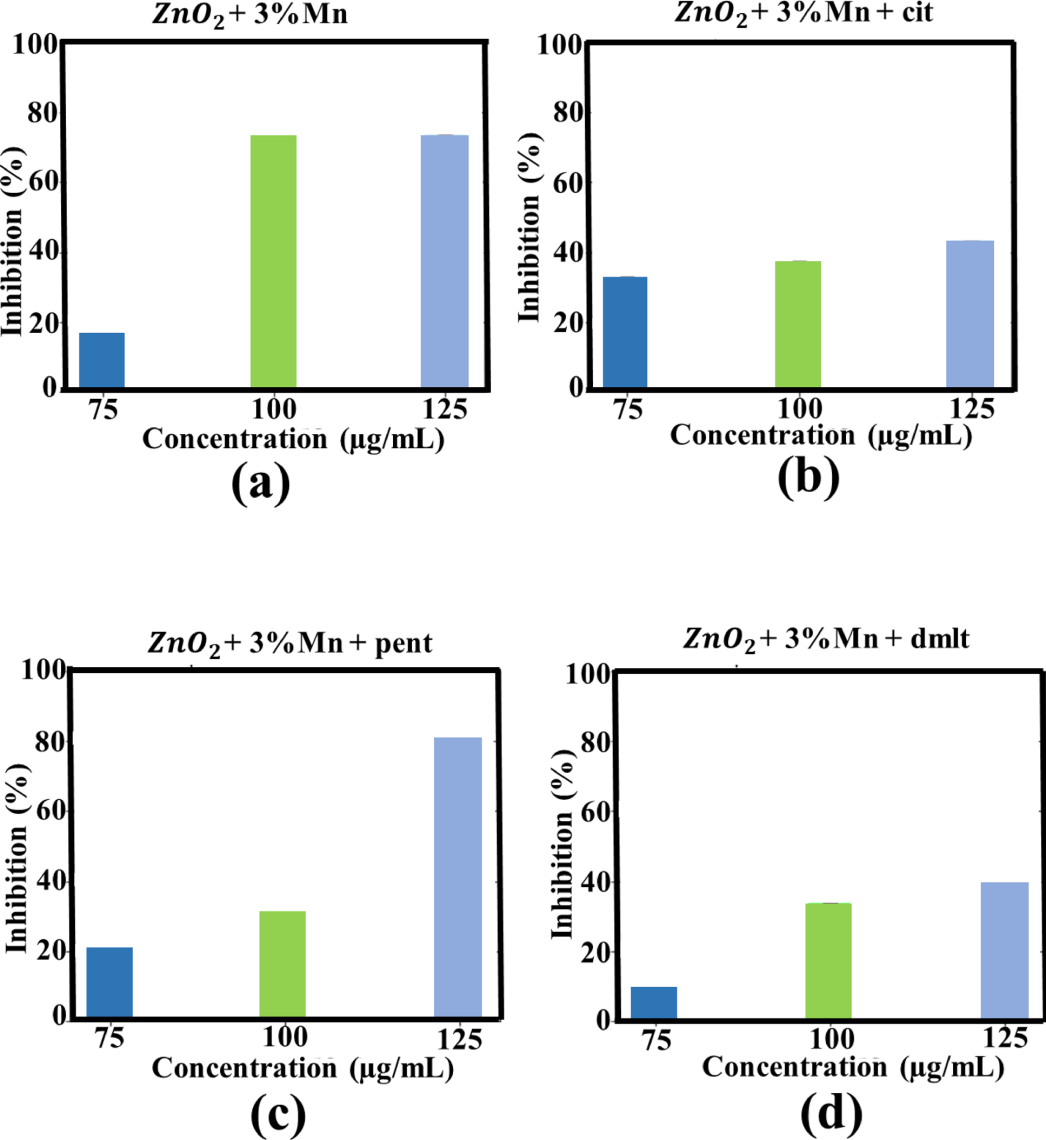

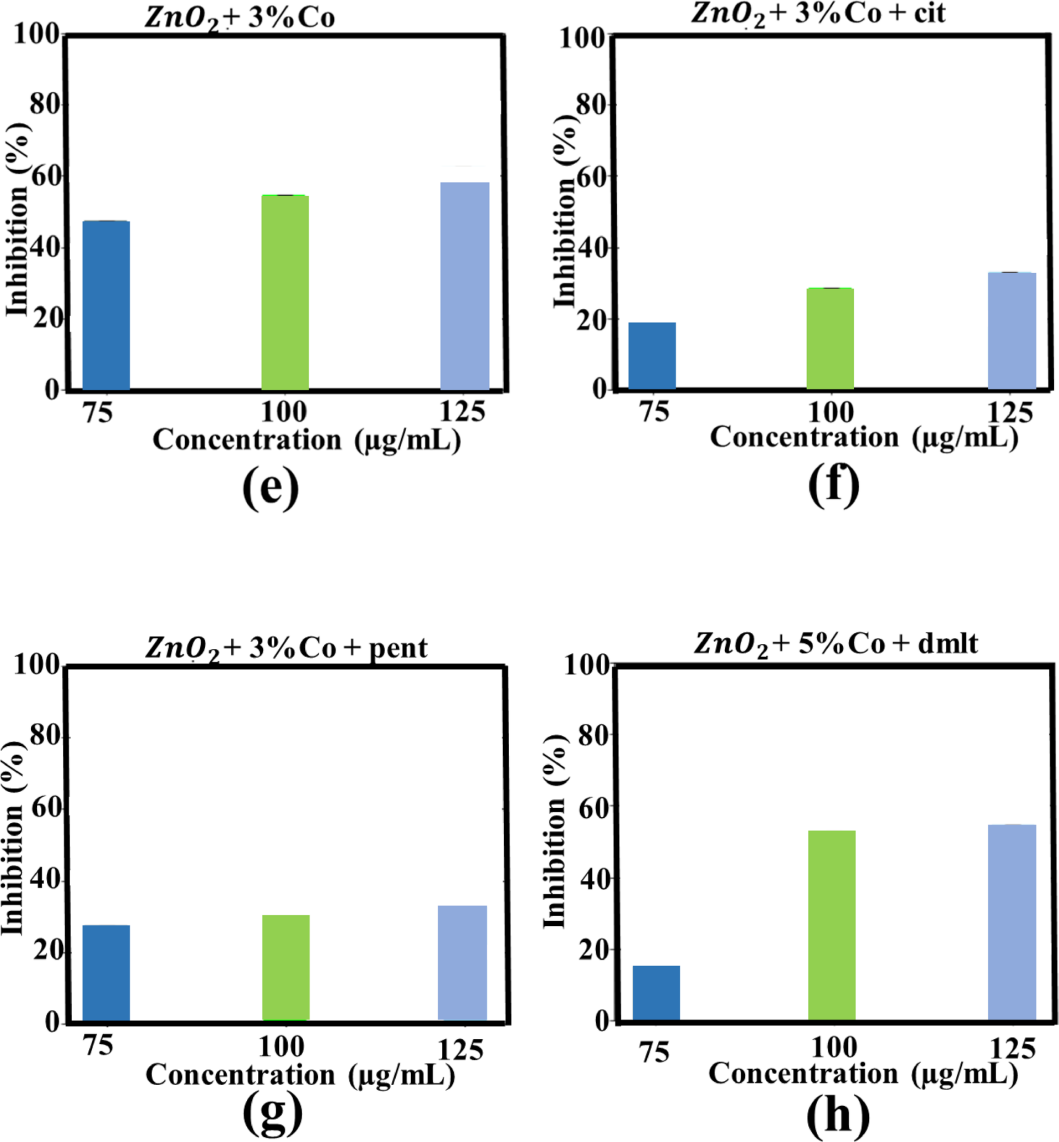
Anti-AChE activities of 3% Mn doped ZnO_2_ NPs (**a**), cit-capped 3% Mn doped ZnO_2_ NPs (**b**), pent-capped 3% Mn doped ZnO_2_ NPs (**c**), dmlt-capped 3% Mn doped ZnO_2_ NPs (**d**), 3% Co doped ZnO_2_ NPs (**e**), cit-capped 3% Co doped ZnO_2_ NPs (**f**), pent-capped 3% Co doped ZnO_2_ NPs (**g**), and dmlt-capped 5% Co doped ZnO_2_ NPs.

### Anti-AChE activity

A progressive and irreversible neurological condition called Alzheimer’s disease (AD) accounts for 60–80% of dementia cases globally. Under the clinical condition of AD, cognitive functions such as memory, executive function, visual-spatial skills, personality, and language impairs gradually^49,50^. The unusual increase in AChE activity in the brain is a key contributor to AD. Various AChE inhibitors, including synthetic and natural compounds, have been explored for effective inhibition of AChE activity.

In the current study, AChE inhibition was carried out by pure ZnO_2_ NPs (ligand capped), Mn-doped and Co-doped ZnO_2_ NPs (with and without ligand molecule) at different concentrations (75, 100 and 125 µg/ml). At highest concentration, cit-, pent-, and dmlt-capped ZnO_2_ NPs reported 75.5 ± 0.1%, 57.5 ± 0.3%, and 41.1 ± 0.2% AChE inhibition, respectively. In case of 3% Mn-doped ZnO_2_ NPs without capping agent and capped with cit-, pent-, and dmlt- exhibited maximum AChE inhibition of 73.2 ± 0.2%, 43.3 ± 0.1%, 82.0 ± 0.3%, and 39.6 ± 0.2%, respectively. In contrast, 3% Co-doped ZnO_2_ NPs and dmlt-capped 5% Co-doped ZnO_2_ NPs exhibited modest inhibition of 62.4 ± 0.3% and 54.5 ± 0.2%, respectively. The results confirmed that the maximum AChE inhibition (82 ± 0.3%) was reported in case of 3% Mn-doped ZnO_2_ NPs with pent as a stabilizer, as shown in Figs. 11 and 12. The direct interaction of ZnO_2_ NPs where the NPs physically bind to the active site of AChE enzyme, lead to the disruption of its activities which is further explored via molecular docking. Another possibility is the generation of ROS species that interact with AChE, causing structural changes in its active site. The enzyme’s ability to break down acetylcholine is affected by such alternations, leading to enhance acetylcholine level inside the brain^51,52^. The solution containing only the assay mixture without NPs was used as the –ve control, serving as a baseline to measure AChE activity in the absence of any inhibitory material and showed no detectable activity^53^. 75, 100, 125 µg/ml concentrations were chosen to enable comparative evaluation with prior studies. Similar dose range were found biological active in AChE inhibition assays in our previous studies^4^. However the current study uses ZnO_2_ NPs with higher oxidative potential.

The results (Figs. 8 and 9; Table 1 and “Growth kinetics of ZnO_2_ NPs and cit-capped ZnO_2_ NPs”) clearly indicating influence of dopant and ligand molecules on biological activities and stability. For instance, cit capped, Co-doped ZnO_2_ NPs exhibited the strongest antibacterial effect (12.5 ± 2.0 mm at 1000 µg/mL) against MRSA, suggesting the synergistic effect of Co-doping and the stabilizing via cit molecules^54^. On the other hand, Mn-doped ZnO_2_ NPs capped with dmlt were more effective (12.3 ± 1.9 mm) against BC, pointing a ligand-mediated improvement in bacterial membrane interaction. For AChE inhibition, the highest activity was observed in Mn-doped with pent capping, pointing that pent molecules on the surface of NPs may offer better surface area compared to others ligands. Overall, cit molecules on the surface of NPs made them more stable, but sometimes compromise on bioactivity. On the other hand, dmlt- and pent- capped systems presented better biological activity in specific cases at the expense of some stability. These comparisons help the critical role of ligand identity and dopant type in tuning ZnO_2_-based NPs for targeted biomedical applications.

### Molecular docking

The antimicrobial and anti-AChE potential of ZnO_2_ NPs was further revealed through molecular docking using Auto-Dock Vina. Two target proteins were selected, including phenol-soluble modulins alpha2 (PSMα2) protein (a crucial virulence component of MRSA) and phospholipase C Regulator (PlcR) (transcriptional regulation in BC). In AChE inhibition, the crystal structure of 1EEA (acetylcholinesterase from *Electrophorus electricus* (electric eel)) were obtain from Protein Data Bank.

**Fig. 13:**
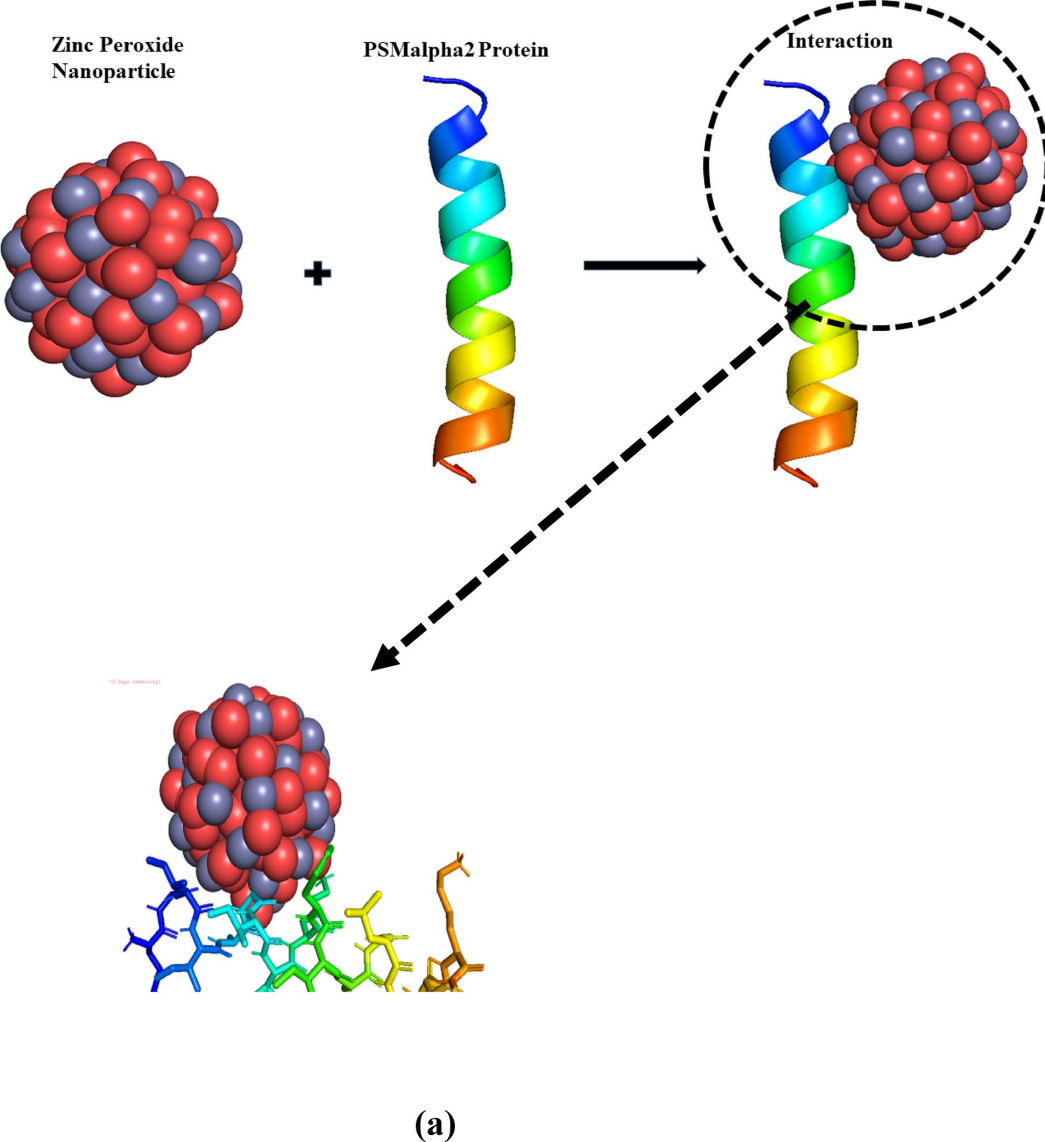

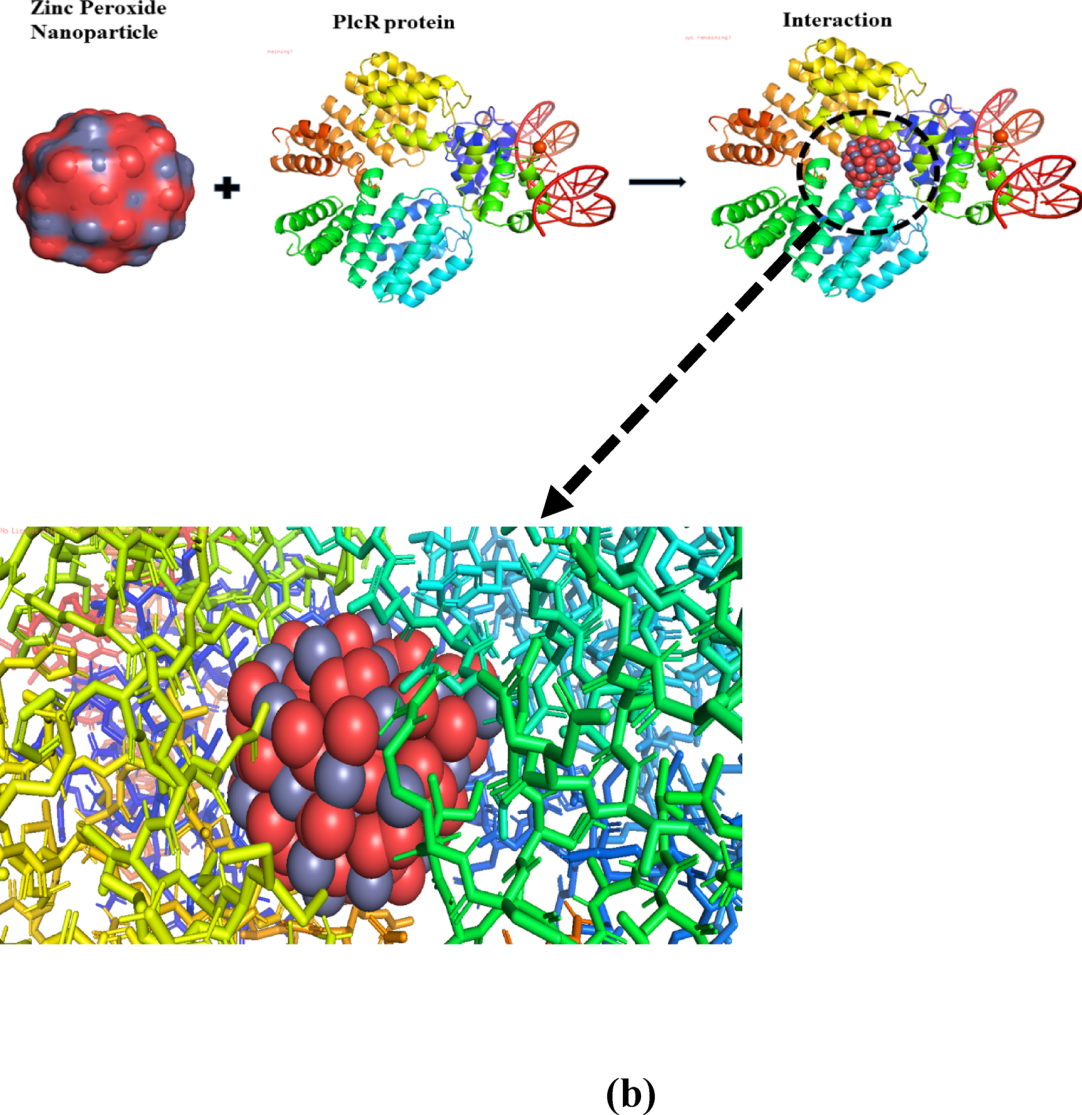

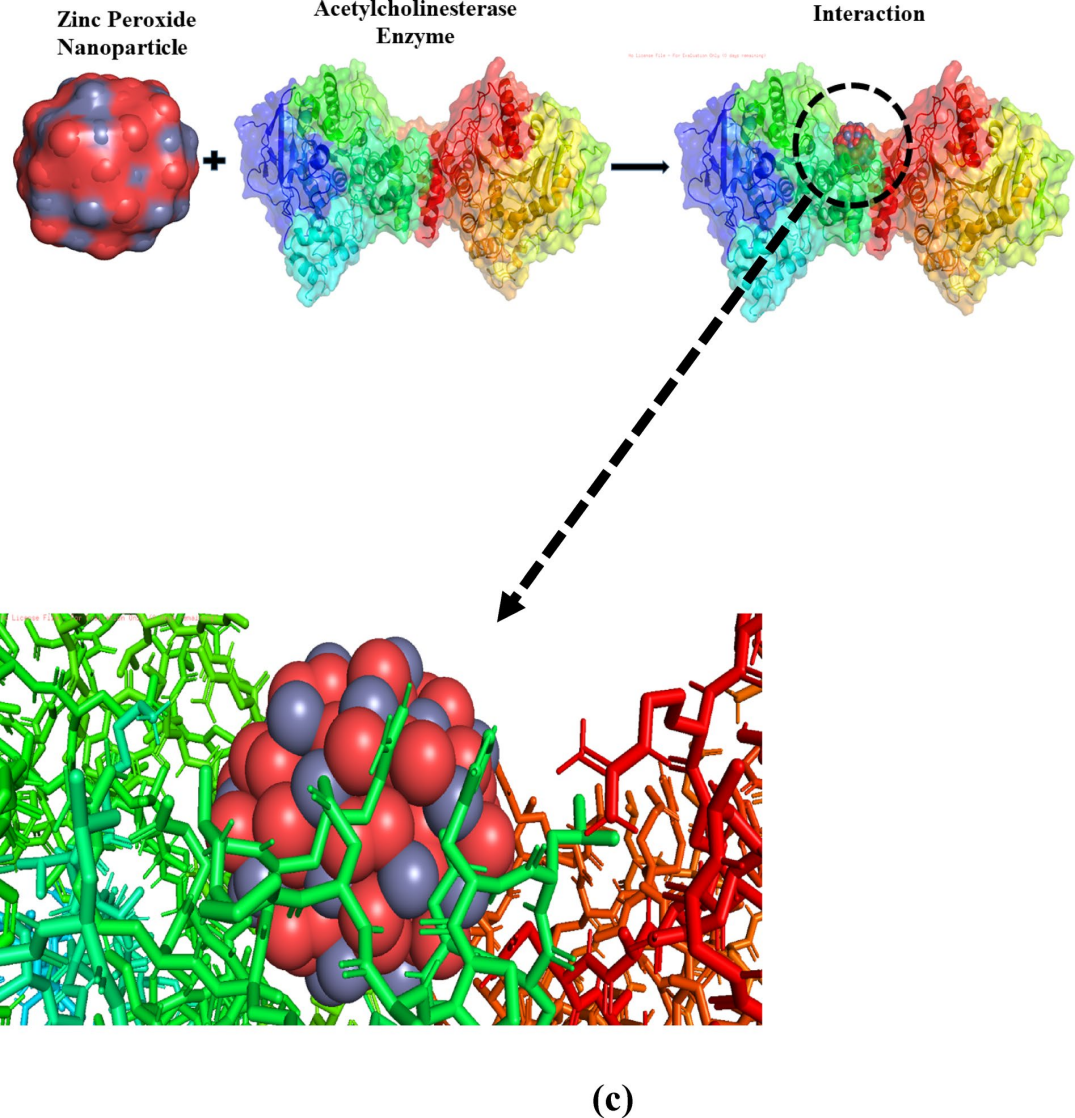
Molecular docking of ZnO_2_ NPs with PSMα2 protein (**a**) PlcR protein (**b**) and 1EEA enzyme (**c**).

Molecular docking targets were selected based on their critical roles in the virulence and pathogenicity of the studied organisms. PSMα2, a MRSA specific cytolytic peptide produced by Staphylococcus aureus, known for disrupting host cell membranes, immune evasion, and biofilm formation. Its membrane-active nature makes it ideal candidate to assess the potential of ZnO2 NPs anti-virulence potential against MRSA^55^. The study indicated a significant interaction between ZnO_2_ NPs and the amphipathic α-helical structure of PSMα2 and demonstrates a binding affinity, which is essential for membrane destabilization. The lowest (best score) and highest binding energies of − 6.1 kcal/mol and − 5.3 kcal/mol were observed upon interaction. The result indicated various kinds of possibilities (interactions) between ZnO_2_ NPs and PSMα2 protein, including hydrogen bonding, hydrophobic interaction, electrostatic interaction and π–π stacking. The hydroxyl groups (confirmed from FT-IR analysis) or oxygen species on the surface of ZnO_2_ NPs interacted with glycine (Gly) (Gly2, Gly6) residues through hydrogen bonding, which consisted of two carbon atoms, five hydrogen atoms, one nitrogen atom, and two oxygen atoms. Such interaction promoted strong intermolecular connectivity. Isoleucine (Ile3, Ile7) and alanine (Ala5) ((consisting of a central carbon atom, a carboxyl group (–COOH), an amino group (–NH_2_), and a side chain (R)) (hydrophobic residues) interacted with NPs via Van der Waals forces. Lysine (Lys9) (with a primary amine group (–NH_2_) and a carboxyl group (–COOH)) interacted via electrostatic force with the positive Zn^2+^ ions on the surface of NPs is responsible for the structural integrity of the binding. Furthermore, the carboxylate group of glutamic acid (Glu16) (two carboxyl groups (–COOH) and one amino group (–NH_2_)) can engage with Zn^2+^ sites and promote coordination bonding. Residues with aromatic side chain like phenylalanine (Phe10) may take part in π–π stacking or van der Waals interaction. The intricate binding mechanism between NPs and proteins is highlighted by these diverse interactions, as shown in Fig. 13a.

Phospholipase C Regulator (PlcR) plays an important role in the expression of virulence factors, including phospholipases, hemolysins, and enterotoxins, which are essential for the pathogenicity of the bacterium and act like a control switch for certain enzymes (Phospholipase C (PLC)) in Gram positive bacteria, especially in BC. PlcR manages the control of these enzymes, specifically in two scenarios, either the bacteria are under stress or trying to cause an infection. Bacteria can invade others cells (human or animals) only when these enzymes are activated^49,50^. The interaction of ZnO_2_ NPs with PlcR protein was reported inferring binding energies ranging from − 18.2 to − 17.0 kcal/mol, indicative of strong interaction. The negatively charged residues, including Glu65, Glu192, Glu193, Asp80, and Asp190, form coordination interactions with Zn^2+^ ions in ZnO_2_ NPs. The O^2−^ atoms in ZnO_2_ NPs enhanced contact stability by establishing 2.7–3.2 Å hydrogen bonds with polar residues such as Lys76, Lys79 and Asn191. The negatively charged regions near Zn^2+^ (due to polarization effects) in ZnO_2_ NPs interacted with positively charged residues including Asp190 and Lys153 as observed at distance of 3.5–4.3 Å. Hydrophilic and hydrophobic areas of PlcR, including hydrophobic residues like Leu188, IIe60, IIe161, and IIe229, interacted with ZnO_2_ NPs through Van der Waals forces. Some other interactions with residues like Pro59, His62 and His189 were also reported. Such complex interactions damage PlcR’s functional domains. By decreased PlcR activity and prevention of various virulence processes mediated by this protein, ZnO_2_ NPs may lessen the pathogenicity of BC, as shown in Fig. 13b.

Furthermore, the acetylcholinesterase enzyme from 1EEA was selected because of its well-characterized crystal structure and frequent use in preliminary in vitro and in silico AChE inhibition studies, having high similarity to human AChE^56^. 1EEA interaction with ZnO_2_ NPs were explored for binding affinity and potential inhibitory mechanism via molecular docking. The following amino acids were found to interact with ZnO_2_ NPs: His381, His387 Tyr382, Asp384, Arg393, Ser203, Glu396, Ala397, Ala528, Ala526, Asp404, Arg525, Gln527, and Cys529, indicating a favorable and complex interaction with the lowest and highest binding energy of -6.5 kcal/mol and − 2.7 kcal/mol through 9 different poses. The key interactions including coordination of Zn^2+^ and O^2−^ ions from ZnO_2_ NPs with negatively charged residues (Glu396, Asp384 and Asp404) and polar residues (Ser203, Tyr382, and Gln527) contributed to the structural stabilization by blocking the active site at enzyme. Positive charged residues (Arg393, Agr525 and Lys) and Zn^2+^ ions were observed at distance of 3.5–4.3 Å along with Ala397, Ala528, Ala526, Ile287 and Leu289 (hydrophobic residues) interaction through van der Waals interactions with NP surfaces. Cysteine (Cys529) could form disulfide bonds (covalent bond formed between the sulfur atoms of two cysteine amino acids within a protein), adding to the stability of the interaction. The results suggested that ZnO_2_ NPs can disrupt AChE functionality, highlighting their potential as AChE inhibitors, as shown in Fig. 13c. The interaction between NPs and protein/enzyme are less explored via molecular docking, particularly for ZnO_2_ system. Kirichenko et al. (2025) used molecular docking to explore the interaction between ZnO NPs and liver proteins, revealing stable complexes and size-dependent binding with focus on general toxicity^57^. In contrast, our work investigates functionalized ZnO_2_ NPs and their interaction with virulence and neuronal protein/enzyme.

## Conclusion

ZnO_2_ and Mn–, Co-doped ZnO_2_ NPs were synthesized through environment-friendly, less toxic, and cost-effective approaches. The samples were characterized using complementary characterization techniques and their potential applications. Single phase crystallization was achieved for all samples. Ligands decoration not only improve the stability but also improve optoelectronic, optical and chemical potential of the synthesized NPs. DFT calculations revealed the adsorption behavior of cit molecules on the ZnO_2_ surface, highlighting atomic rearrangements and Zn–O bond formation. Additionally, *in-situ* growth kinetics studies confirmed that cit capping stabilized ZnO_2_ NPs, preventing excessive growth and agglomeration. The antibacterial assay exhibited moderate and strong antimicrobial activity against *Methicillin-resistant Staphylococcus aureus* and *Bacillus cereus*, respectively. The results were further validated via molecular docking. The 3% Mn-doped ZnO_2_ NPs without any ligand molecule, cit and dmlt-capped were found active against Gram + ve bacteria. Co-doped ZnO_2_ NPs decorated with cit molecules exhibited a striking trend of direct increase in antimicrobial efficiency with the increase of concentration but remains inactive against *Bacillus cereus*. On the other hand, the potential for pure and doped ZnO_2_ NPs as anti-AChE agents were explored. The investigations reported that these ultra-small NPs exhibited remarkable potential to inhibit AChE activity, highlighting important implications and firmly positioning the synthesized NPs as a promising candidate with considerable therapeutic potential in Alzheimer’s disease (AD) therapy.

### Strengths, limitations, future perspectives, and SDG relevance

The current present’s synthesis of uncapped and organic ligand capped of pristine ZnO_2_ and doped (Mn–/Co–) ZnO_2_ NPs. The full picture of the study including structural, electronic, growth kinetics, and biological properties, we used both experimental and theoretical method, such as XRD, FTIR, UV–Vis, DFT calculations, and molecular docking. The results show ligand-assisted stability and increased antibacterial and anti-AChE activities show that these NPs could be consider in treating antimicrobial resistance and Alzheimer’s disease. While the study shows promising biological activities, it is limited to in vitro evaluations. Further validation via in vivo and clinical studies are required. Additionally, more advance simulation techniques are needed for deeper insights and long-term assessments. Other dopant (transition metals) and organic ligands could also be explored and understand their impact on biocompatibility and selectivity towards specific bacterial strains (gram + ve and gram –ve) and enzymes. This study aligns with the United Nations Sustainable Development Goals (SDGs), particularly:

- SDG 3 (Good Health and Well-being)—The study contributes to novel antimicrobial and neuroprotective solutions, addressing drug-resistant infections and neurodegenerative diseases.
- SDG 6 (Clean Water and Sanitation)—Potential use in water disinfection due to antibacterial properties.
- SDG 9 (Industry, Innovation, and Infrastructure)—Development of eco-friendly nanomaterials for medical and environmental applications.
- SDG 12 (Responsible Consumption and Production)—Green synthesis methods reduce hazardous waste, promoting sustainable nanomaterial production.
- SDG 13 (Climate Action)—Eco-friendly synthesis minimizes carbon footprint compared to traditional chemical routes.

## Supplementary Information

Below is the link to the electronic supplementary material.

Supplementary Material 1

## Data Availability

All data generated or analysed during this study are included in this published article and its supplementary information files.
