# Supplementary material for: Ligand-controlled growth and stabilization of doped ZnO2 nanoparticles for dual antibacterial and enzyme inhibition
Source: Sci Rep. 2025 Aug 16;15:29994. doi: 10.1038/s41598-025-15917-6 (PMC12357922; doi:10.1038/s41598-025-15917-6)
Supplement: Supplementary file 1 — Supplementary Material 1 [file 41598_2025_15917_MOESM1_ESM.docx]

Supporting Information

# **Nucleation and Stabilization of ZnO_2_ and Doped (Mn, Co) ZnO₂ Nanoparticles: Potent Antimicrobial Agents and Acetylcholinesterase Inhibitors**

**Imran Ullah^a*^,** **Reinhard B Neder^a*^, Huma Parwaz^b^, Zul Kamal^c,d^, Cai-Hong Zhan^e,*^, Komal Qazi^f^, Inam ud din^g^, Hari Pokhrel^h^**

^a^Institute of Condensed Matter Physics, Chair of Crystallography and Structural Physics, Friedrich-Alexander University, Staudt Street 3, 91058 Erlangen, Germany.

^b^Department of Physics, University of Science and Technology Bannu, KPK, Pakistan

^c^Department of Pharmacy, Shaheed Benazir Bhutto University, Sheringal, 18300, Dir Upper, Khyber Pakhtunkhwa, Pakistan.

^d^Chongqing University of Chinese Medicines, Puguobao Road, 402760, Chongqing, P.R China.

^e^Key Laboratory of the Ministry of Education for Advanced Catalysis Material, College of Chemistry and Materials Science, Zhejiang Normal University, Jinhua 321004, China.

^f^Department of Biotechnology, University of Science and Technology Bannu, Bannu, KPK, Pakistan.

^g^Department of Physics University of Peshawar, Peshawar, Khyber Pakhtunkhwa, Pakistan.

^h^Department of Physics, Friedrich-Alexander University, Erlangen, Germany.

***Corresponding authors:**

**Imran Ullah (IU)** Email: [imran.ullah@fau.de](mailto:imran.ullah@fau.de), [imranwazir83@gmail.com](mailto:imranwazir83@gmail.com)

**Reinhard B. Neder (RN)** Email: reinhard.neder@fau.de

**Cai-Hong Zhan (CZ)** Email: chzhan@zjnu.cn

**Co-authors**

**Huma Parwaz (HP)** Email: humaparwaz99@gmail.com

**Zul Kamal (ZK)** Email: [xulkamal@sbbu.edu.pk](mailto:xulkamal@sbbu.edu.pk)

**Komal Qazi (KQ)** Email: komalqazi16@gmail.com

**Inam Ud Din (ID)** Email: malikinam.icp@gmail.com

**Hari Pokhrel (HP)** Email: hari.pokhrel@fau.de

**1.0 Chemicals used during synthesis**

For the synthesis of nanoparticles (NPs), the chemicals were either bought from Sigma Aldrich or Roth. The chemicals were used without further purification or any additional treatment and tabulated in **Table S1**.

Table S1 Details of Chemicals used during Synthesis

| **Name** | **Chemical Formula** | **CAS** | **Purity** | **Provider** |
| --- | --- | --- | --- | --- |
| Zinc Acetate Dihydrate | ${({CH}_{3}COO)}_{2}$∙$Zn\cdot2\cdot H_{2}O$ | 5970-45-6 | ≥ 99% | Sigma Aldrich/Roth |
| Tetra-methyl ammonium Hydroxide (25% in methanol) | ${{(CH}_{3})}_{4}N(OH)$ | 75-59-2 | $\approx$88% | Sigma Aldrich |
| Ammonium Hydroxide (25% in water) | ${NH}_{4}OH$ | 1336-21-6 | ≥ 99% | Sigma Aldrich |
| Hydrogen Peroxide (30% in water) | $H_{2}O_{2}$ | 7722-84-1 | ≥ 99% | Roth |
| Manganese acetate tetra Hydrate | $\left( {CH}_{3}COO \right)_{2}\cdot Mn\cdot4H_{2}O$ | 6156-78-1 | ≥ 99% | Sigma Aldrich |
| Cobalt Acetate tetra Hydrate | $\left( {CH}_{3}COO \right)_{2}\cdot Co\cdot4H_{2}O$ | 6147-53-1 | ≥ 99% | Sigma Aldrich |
| Citric Acid | $HOC\left( COOH \right)\cdot{({CH}_{2}COOH)}_{2}$ | 77-92-9 | ≥ 99.5% | Sigma Aldrich |
| 1,5-diphenyl-1,3,5-pentanetrione | $C_{6}H_{5}COCH_{2}$  $\cdot COCH_{2}COC_{6}H_{5}$ | 1467-40-9 | ≥ 98% | Sigma Aldrich |
| Dimethyl-L tartrate | ${\begin{aligned} [CH\left( OH \right) \\ \cdot{CO}_{2}{CH}_{3}] \end{aligned}}_{2}$ | 608-68-4 | 99% | Sigma Aldrich |

The final product were obtained in the form of powder, as shown in the **Fig. S1.**


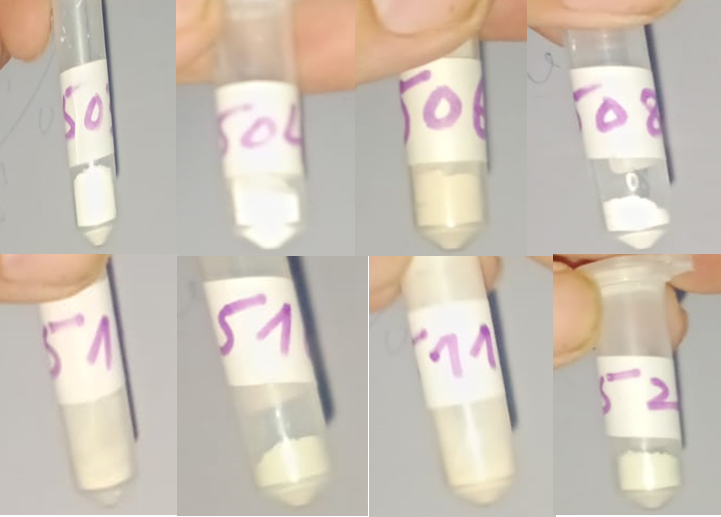


Figure S1 Representative images of the synthesized NPs

Table S2. Description of ZnO_2_-Based NPs Samples with Corresponding Capping Agents and Dopants

| Sample | Capping agent | Dopant |
| --- | --- | --- |
| ZnO_2_ | __ | __ |
| ZnO_2_ cit | Citric acid | __ |
| ZnO_2_ pent | 1,5-diphenyl-1,3,5-pentanetrione | __ |
| ZnO_2_ dmlt | Dimethyl-L tartrate | __ |
| Mn_x_-doped Zn_1-x_O_2_^*^ | Manganese doped ZnO_2_ | If x= 0.03 mean 3% doping  If x = 0.05 mean 5% doping |
| Co_x_-doped Zn_1-x_O_2_^*^ | Cobalt doped ZnO_2_ | If x= 0.03 mean 3% doping  If x = 0.05 mean 5% doping |

^*^Mn-/Co-doped ZnO_2_ NPs were synthesized with and without capping agents (cit-, pent-, and dmlt.)

| Sample | Zinc Acetate (g) | Cobalt Precursor (g) | Manganese Precursor (g) | cit (g) | pent (g) | dmlt (g) | Performed Activity/activities |
| --- | --- | --- | --- | --- | --- | --- | --- |
| ZnO_2_ (pure) | 0.5926 | – | – | – | – | – | MRSA  BC |
| ZnO_2_ cit | 0.5926 | – | – | 0.0018 | – | – | AChE inhibition |
| ZnO_2_ pent | 0.5926 | – | – | – | 0.0025 | – | AChE inhibition |
| ZnO_2_ dmlt | 0.5926 | – | – | – | – | 0.0017 | AChE inhibition |
| 3% Co-doped ZnO_2_ | 0.5926 | 0.0202 | – | – | – | – | AChE inhibition |
| 3% Co-ZnO_2_ + cit | 0.5926 | 0.0202 | – | 0.0018 | – | – | AChE inhibition |
| 3% Co-ZnO_2_ + pent | 0.5926 | 0.0202 | – | – | 0.0025 | – | AChE inhibition |
| 5% Co-ZnO_2_ + cit | 0.5926 | 0.0336 | – | 0.0018 | – | – | MRSA  BC |
| 5% Co-ZnO_2_ + dmlt | 0.5926 | 0.0336 | – | – | – | 0.0017 | AChE inhibition |
| 3% Mn-doped ZnO_2_ | 0.5926 | – | 0.0198 | – | – | – | MRSA  BC  AChE inhibition |
| 3% Mn-ZnO_2_ + cit | 0.5926 | – | 0.0198 | 0.0018 | – | – | AChE inhibition |
| 3% Mn-ZnO_2_ + pent | 0.5926 | – | 0.0198 | – | 0.0025 |  | AChE inhibition |
| 3% Mn-ZnO_2_ + dmlt | 0.5926 | – | 0.0198 | – | – | 0.0017 | MRSA  BC  AChE inhibition |
| 5% Mn-doped ZnO_2_ | 0.5926 | – | 0.0331 | – | – | – | – |
| 5% Mn-ZnO_2_ + cit | 0.5926 | – | 0.0198 | 0.0018 | – | – | – |
| 5% Mn-ZnO_2_ + dmlt | 0.5926 | – | 0.0198 | – | – | 0.0017 | – |

Table S3 Summary of uncapped and organic ligand capped ZnO_2_ and doped ZnO_2_ NPs with precursors and activities performed.

# **2.0 Crystal Structure**

ZnO_2_ has a Pyrite-like structure and crystallizes in a cubic crystal system with the space group Pa-3 (No. 205), where the lattice parameters a = b = c and α = β = γ = 90^°^. Each Zn^2+^ ion is surrounded by six equivalent O^1-^ ion forming ZnO_6_ octahedra, sharing their corners with twelve equivalent ZnO_6_ octahedra and with six equivalent OZn_3_ tetrahedra. The lengths of all Zn˗O bonds in ZnO_6_ octahedra are ≈ 2.14 Å. While O^1-^ ion is surrounded by three equivalent Zn^2+^ and one O^1-^ ions forming OZn_3_O tetrahedra. Their corners are shared with three equivalent ZnO_6_ octahedra and fifteen equivalent OZn_3_O tetrahedra. The bond length between Oxygen atoms in OZn_3_O tetrahedra is ≈ 1.50 Å. The distinct corner-sharing octahedral, and tetrahedral combination with different bond lengths lead to various unique properties, shown in the **Fig. S2** [1].


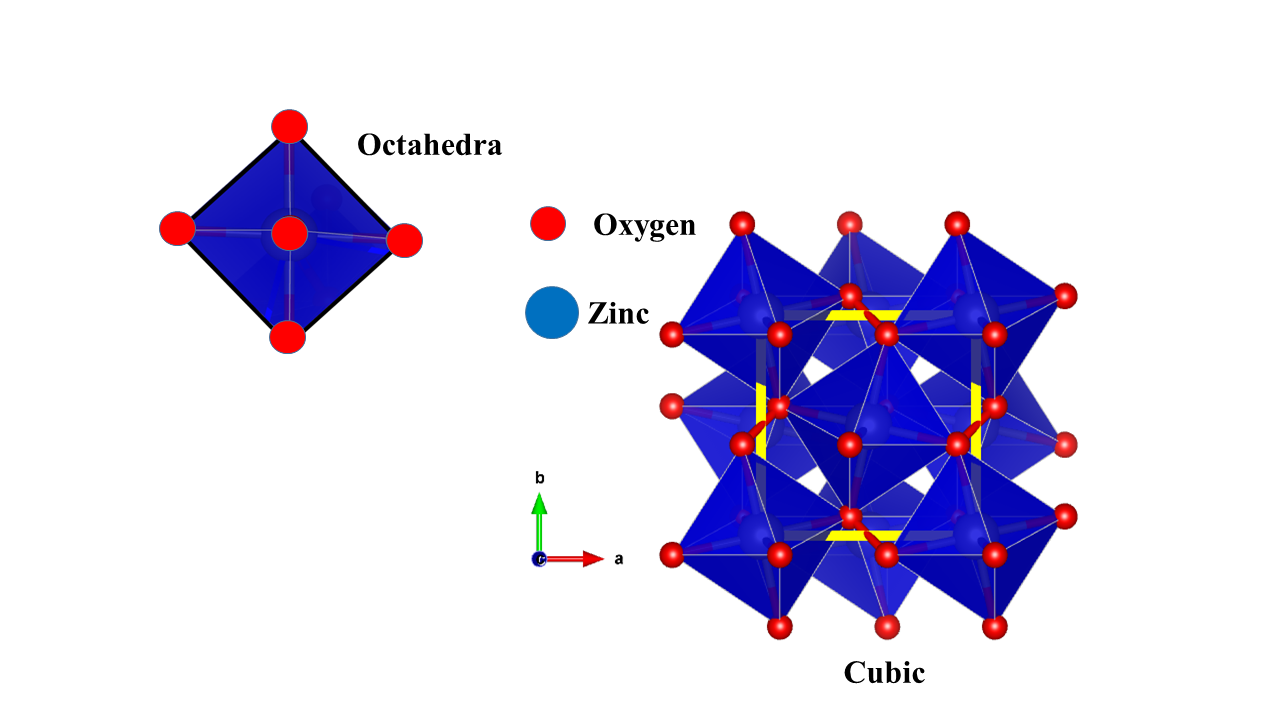


Figure S2 Crystal Structure of Zinc Peroxide (ZnO_2_)

# **3.0 Energy minimization workflow for molecular docking**

Prior molecular docking, the designated protein/enzyme and ligand (ZnO_2_ NPs) were prepared using UCSF Chimera. Initially, water molecules and non-essential ligands were eliminated, as the majority of crystallographic structures (protein/enzyme) include water molecules from the crystallization process that lack biological significance. Although water does not engage directly in the binding interactions. It is vital to eliminate non-essential ligands as they may obstruct the active binding sites. Protein structures frequently lack hydrogens due to the limitations of X-ray crystallography in resolving them. Polar hydrogen was incorporated and is essential for delineating the molecule electrostatic potential and binding interactions. Kollman charges were included into the protein to represent its electrostatic environment, which is crucial for precise docking score calculations. The AMBER ff14SB force field was employed to assign charges and perform energy minimization utilizing 500 steps of steepest descent followed by 10 steps of conjugate gradient. The CIF file for the ligand was acquired from the Materials Project. The unit cell was optimized through the SIESTA code. The optimized unit cell was extended along a, b, and c directions using DISCUS Suite [7]. The extended structure was trimmed to an appropriate shape. AutoDock tool was utilized to create the configuration file for protein and ligand interaction. Vina software was employed to investigate the interaction between ligand and protein [8].

## **4.0 Formation of ZnO_2_ US-NPs**

At the first stage zinc acetate dihydrate (Zn(CH_3_COO)_2_∙2H_2_O) dissociates into zinc ions (Zn^2+^) and acetate ions ((CH_3_COO)^-^), releasing water (H_2_O) molecules. Protonation of acetate occurs through water molecules, resulting acetic acid and hydroxide ions (OH^˗^). At the same time, NH_4_OH provide ${NH}_{4}^{+}$ and ${OH}^{-}$, as reported by Imran Ullah et. al [6].

$${Zn({CH}_{3}COO)}_{2}\cdot2H_{2}O\to{Zn}^{2+}+{2\left( {CH}_{3}COO \right)}^{-}+2H_{2}O$$

${{CH}_{3}COO}^{-}+H_{2}O\to{CH}_{3}COOH+ {OH}^{-}$

${NH}_{4}OH\to{NH}_{4}^{+}+{OH}^{-}$

The solution has a high concentration of ${OH}^{-}$ radicals and reacts with ${Zn}^{2+}$ by forming ${Zn(OH)}_{2}$or${Zn(OH)}_{4}^{2-}$. While ammonia reacts with ${Zn}^{2+}$ and form${[{Zn({NH}_{3})}_{4}]}^{2+}$.

${Zn}^{2+}+2{OH}^{-}\to{Zn(OH)}_{2}$

${Zn}^{2+}+4{NH}_{3}\to{[{Zn({NH}_{3})}_{4}]}^{2+}$

${Zn(OH)}_{2}$ or ${Zn(OH)}_{4}^{2-}$ further react with H_2_O_2_ to form ZnO_2_ US-NPs along with the release of a water molecule and hydroxyl radical.

${Zn(OH)}_{2}+H_{2}O_{2}\to{ZnO}_{2}+{2H}_{2}O$

${Zn(OH)}_{4}^{2-}+H_{2}O_{2}\to{ZnO}_{2}+{2H}_{2}O+2{OH}^{-}$

${[{Zn({NH}_{3})}_{4}]}^{2+}$can react with ${OH}^{-}$ that results in formation of ZnO NPs. By oxidizing ZnO NPs through $H_{2}O_{2}$ and results in the formation of ZnO_2_ NPs.

${[{Zn({NH}_{3})}_{4}]}^{2+}+ 2{OH}^{-}\to ZnO+4{NH}_{3}+H_{2}O$

$ZnO+H_{2}O_{2}\to{ZnO}_{2}+H_{2}O$

## **5.0 XRD of Mn- and Co-dope ZnO_2_ NPs**


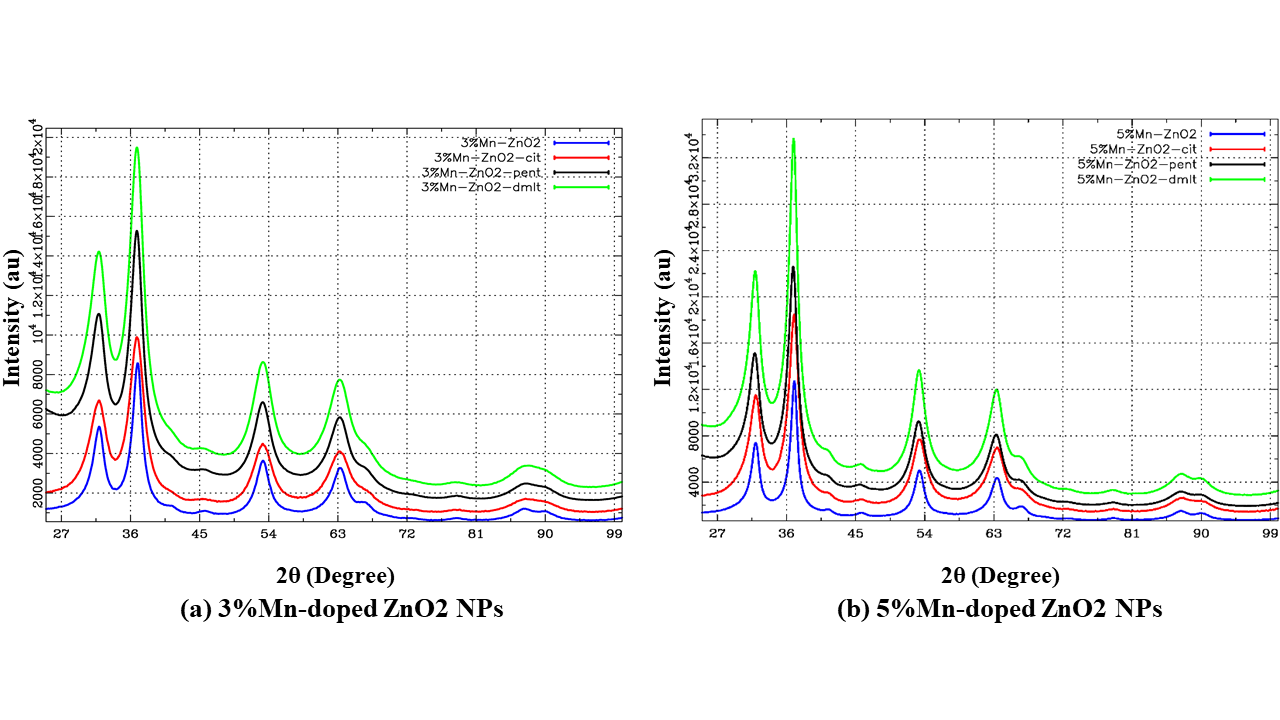


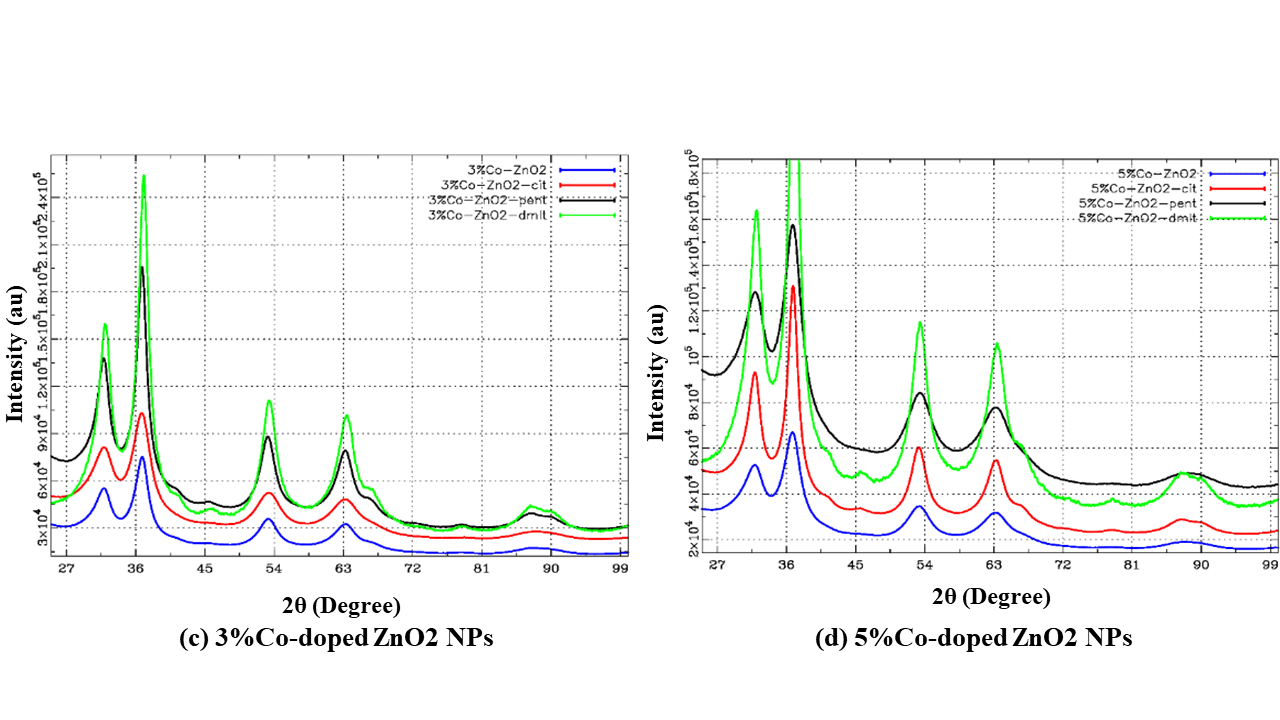


Figure S3 XRD of 3%, 5% Mn-doped ZnO_2_ and Co-doped ZnO_2_ NPs with and without ligand molecules capping

## **6.0 Structural Studies**

Details information about the structural studies is provided in the main manuscript at **section 2.3, 3.2, and 3.3.** The impact of doping on peak position from various crystallographic planes are tabulated in the **Table S4, S5**.

Table S4 Pure, 3%, and 5% Mn-doped ZnO_2_ NPs and corresponding peak positions

| Peak | Peak Position of ZnO_2_ | Peak Position of  3% Mn-doped ZnO_2_ | Peak Position of  5% Mn-doped ZnO_2_ |
| --- | --- | --- | --- |
| (111) | 31.792 | 31.816 | 31.800 |
| (200) | 36.822 | 36.830 | 36.780 |
| (220) | 53.140 | 53.140 | 53.140 |
| (311) | 63.100 | 63.150 | 63.220 |

Table S5 Pure, 3%, and 5% Co-doped ZnO_2_ NPs and corresponding peak positions

| Peak | Peak Position of ZnO_2_ | Peak Position of  3% $Co$-doped ZnO_2_ | Peak Position of  5% Co-doped ZnO_2_ |
| --- | --- | --- | --- |
| (111) | 31.792 | 31.817 | 31.660 |
| (200) | 36.822 | 36.855 | 36.570 |
| (220) | 53.140 | 53.140 | 53.140 |
| (311) | 63.100 | 63.209 | 63.050 |


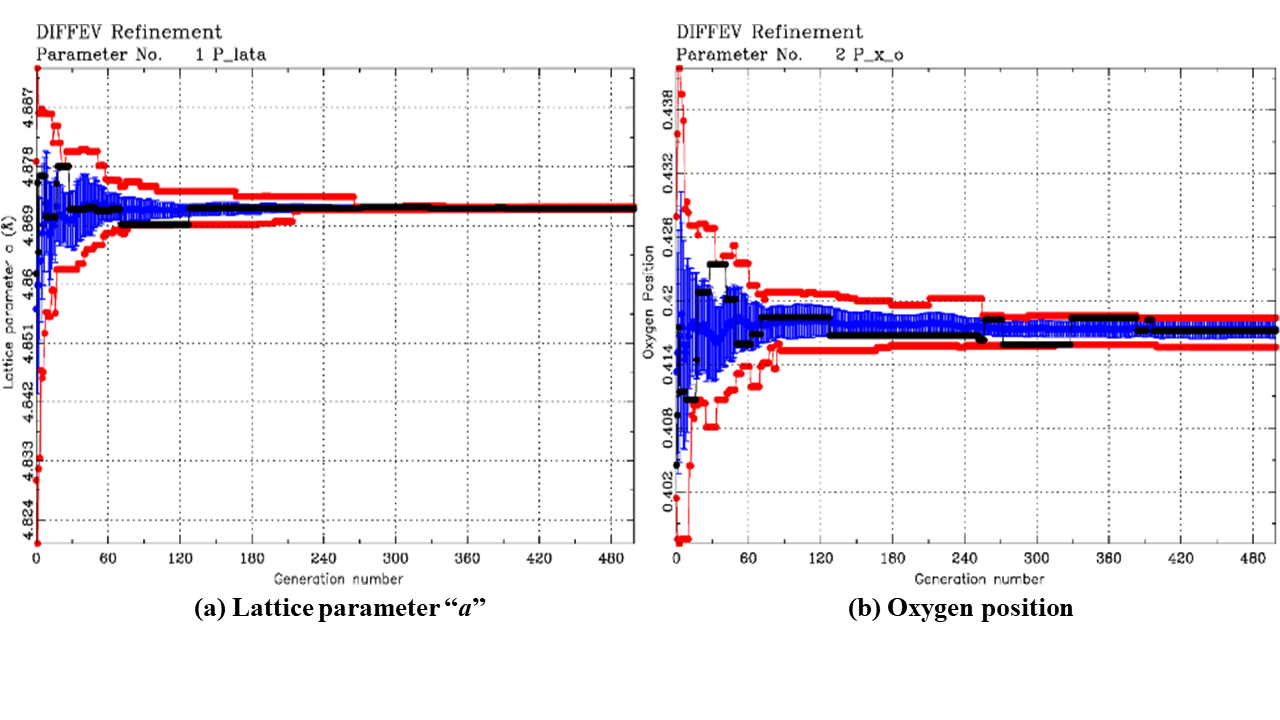


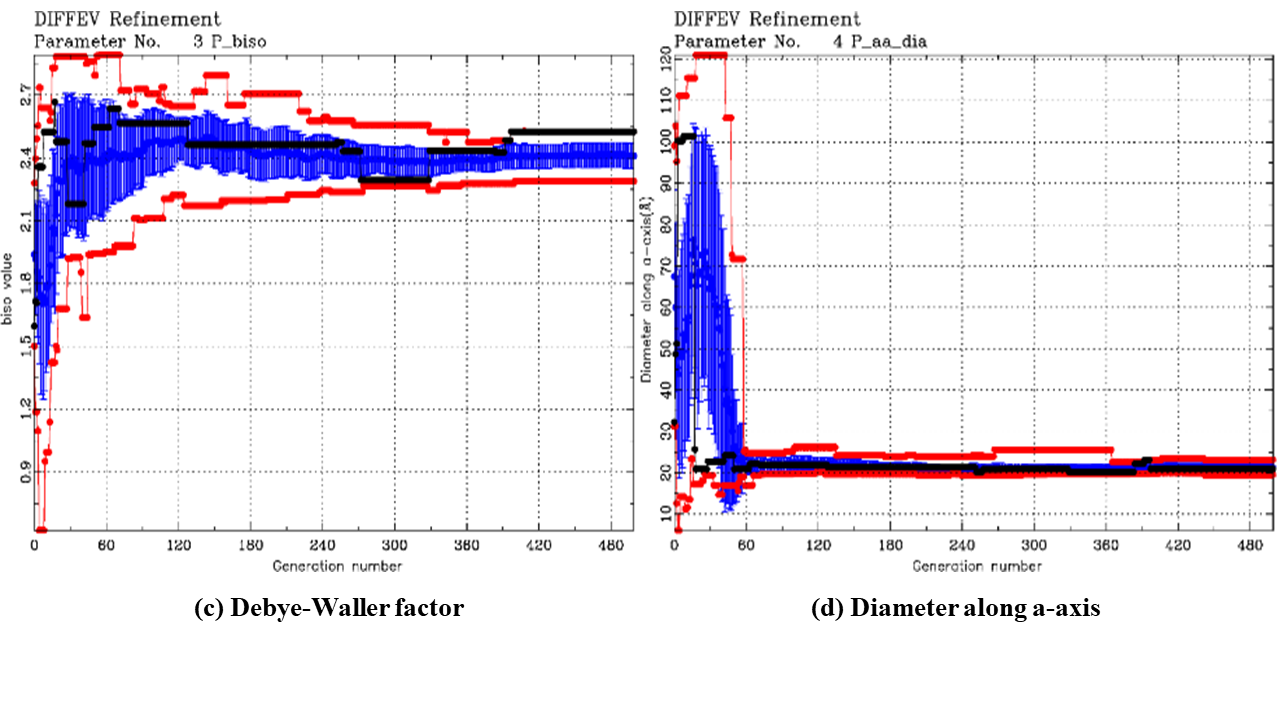


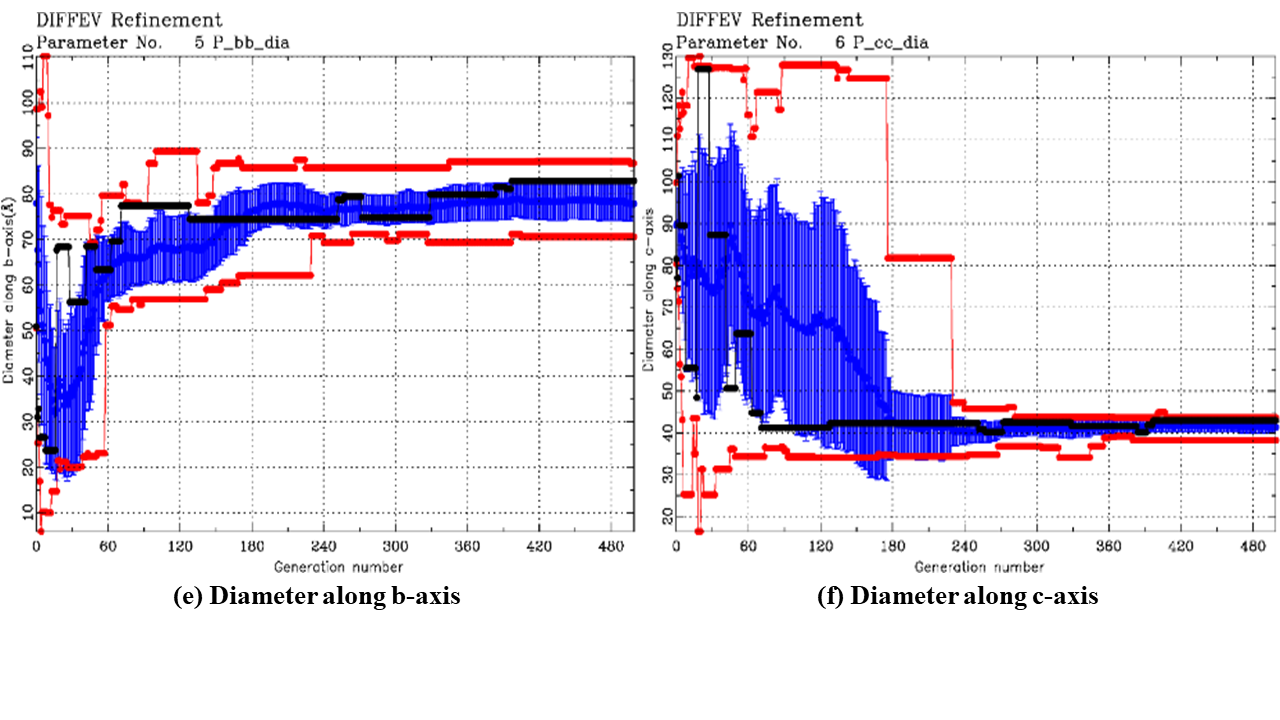


Table S6 Various refine parameters and their corresponding values

Figure S4 Various refine parameters after 500 Generations

| Sample name | P_lata | P_x_o | P_biso | P_aa_dia | P_bb_dia | P_cc_dia |
| --- | --- | --- | --- | --- | --- | --- |
| ${ZnO}_{2}$ | 4.87156 | 0.41755 | 2.71162 | 20.29584 | 69.35012 | 42.55576 |
| ${ZnO}_{2}$ + cit | 4.86108 | 0.41774 | 2.66388 | 18.48511 | 50.18783 | 58.51097 |
| ${ZnO}_{2}$ + pent | 4.86694 | 0.41689 | 2.3861 | 19.62689 | 62.90457 | 31.00815 |
| ${ZnO}_{2}$+ dmlt | 4.87157 | 0.41718 | 2.52559 | 20.93975 | 82.88928 | 42.96803 |
| 3% $Mn$-doped ${ZnO}_{2}$ | 4.86872 | 0.41533 | 2.52793 | 20.40610 | 89.64342 | 41.91932 |
| 5% $Mn$ -doped ${ZnO}_{2}$ | 4.86792 | 0.41640 | 2.78130 | 24.72550 | 113.94889 | 63.54883 |
| 3% $Mn$ -doped ${ZnO}_{2}$ cit | 4.86800 | 0.41824 | 2.64537 | 19.47424 | 52.52982 | 35.58916 |
| 5% $Mn$ -doped ${ZnO}_{2}$ cit | 4.86926 | 0.41844 | 2.78067 | 20.23749 | 86.66782 | 42.24133 |
| 3% $Mn$ -doped ${ZnO}_{2}$ pent | 4.86716 | 0.41980 | 2.68690 | 20.50154 | 57.78474 | 103.19403 |
| 5% $Mn$ -doped ${ZnO}_{2}$ pent | 4.86721 | 0.41639 | 2.89542 | 24.41927 | 75.41504 | 59.21664 |
| 3% $Mn$ -doped ${ZnO}_{2}$ dmlt | 4.86713 | 0.41892 | 2.71918 | 18.78073 | 52.87346 | 111.88283 |
| 5% $Mn$ -doped ${ZnO}_{2}$ dmlt | 4.86723 | 0.41667 | 2.86741 | 19.70436 | 96.94010 | 47.42081 |
| 3% $Co$-doped ${ZnO}_{2}$ | 4.86694 | 0.42372 | 2.61201 | 20.76070 | 58.28842 | 74.29428 |
| 5% $Co$-doped ${ZnO}_{2}$ | 4.86682 | 0.41662 | 2.81323 | 11.43079 | 46.63069 | 97.30734 |
| 3% $Co$-doped ${ZnO}_{2}$cit | 4.85594 | 0.41814 | 2.86518 | 15.18356 | 43.44179 | 129.84234 |
| 5% $Co$-doped ${ZnO}_{2}$cit | 4.86715 | 0.41978 | 2.84406 | 20.06833 | 78.54864 | 43.42960 |
| 3% $Co$-doped ${ZnO}_{2}$pent | 4.86721 | 0.41975 | 2.68584 | 20.69027 | 91.12780 | 47.06603 |
| 5% $Co$-doped ${ZnO}_{2}$pent | 4.86712 | 0.42161 | 2.71043 | 11.63869 | 40.61994 | 122.98474 |
| 3% $Co$-doped ${ZnO}_{2}$dmlt | 4.86880 | 0.41865 | 2.65384 | 20.19547 | 93.29753 | 40.66330 |
| 5% $Co$-doped ${ZnO}_{2}$dmlt | 4.87174 | 0.41966 | 2.71752 | 19.78867 | 82.80544 | 42.16652 |

## **7.0 In-situ study of ZnO_2_ NPs and cit-capped ZnO_2_ NPs**

In situ studies was performed at European Synchrotron Radiation Facility (ESRF) in France (beam ID 15A). **Fig. S5** shows the data sets collected from dissolution of metal salts till formation of NPs. Further details are provided in the main manuscript at **section 3.5.**


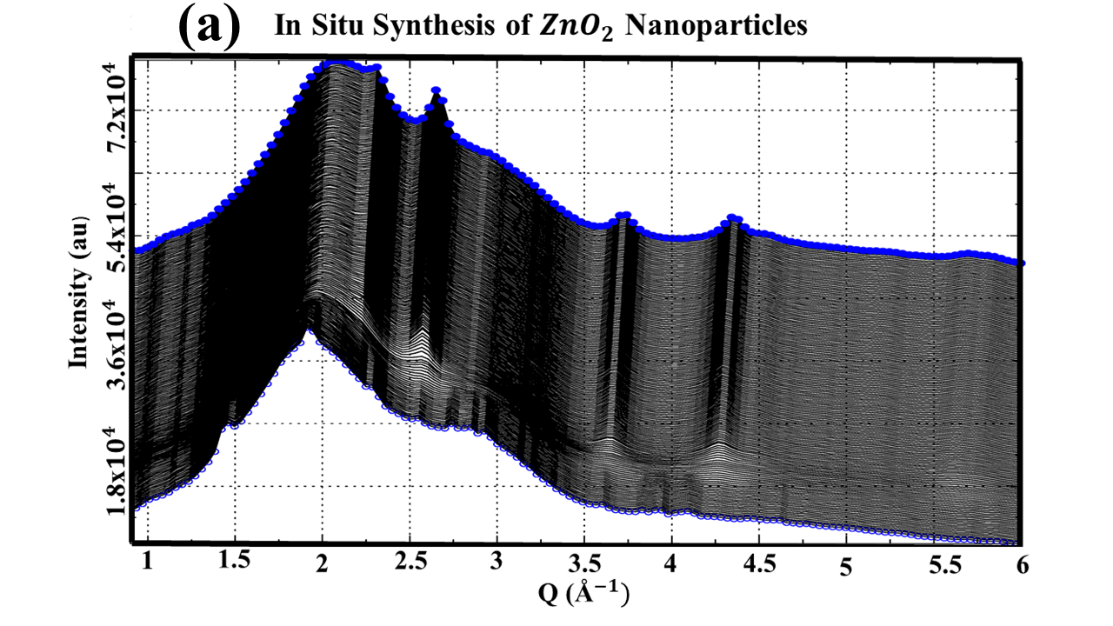


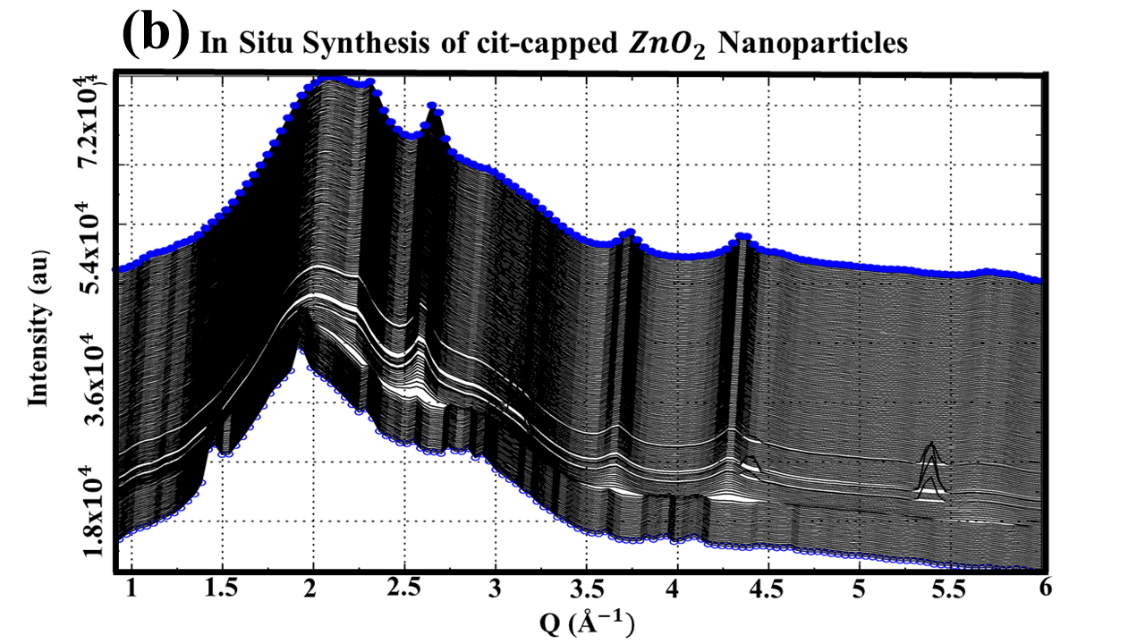


Figure S5 Scan obtain during the *in-situ* study of ZnO_2_ NPs (a) without ligand molecule (b) with cit as capping agent

# **8.0 FT-IR Spectroscopy**

FT-IR of the synthesized sample can be found in **Fig. S6**. Several absorption bands in the finger print region, corresponding to ZnO_2_. The fingerprint region bands at 661.46, 841.22, 941.90, 1010.40, and 1404.08 cm^-1^. Many absorption bands were observed in the functional group region, including 1487.01, 1650.47, 2361.62, and 3351.57 cm^-1^ to gain insight into the functional group present at the surface of ZnO_2_ NPs. The absorption bands at 661.46 cm^-1^, 841.22 cm^-1^, and 941.90 cm^-1^ are characteristic of $Zn-O$ vibration in ZnO_2_ structure while at 1010.40 cm^-1^ and 1404.08 cm^-1^ corresponds to the peroxide $\left( O_{2}^{-2} \right)$ ions of NPs. The band at 1487.01 cm^-1^ are characterized by $C-O$ and $C-C$ bonds because of the presence of citrate/other ligand molecules at the surface NPs. The bands at 2361.62 cm^-1^ and 1650.47 cm^-1^ may be due to the bending vibration of $H-O-H$. The presence of two absorption peaks at 3351.57 cm^-1^ and 2361.62 cm^-1^ shows that water molecules or hydroxyl groups are attached at the surface of the synthesised ZnO_2_ NPs [3-6].


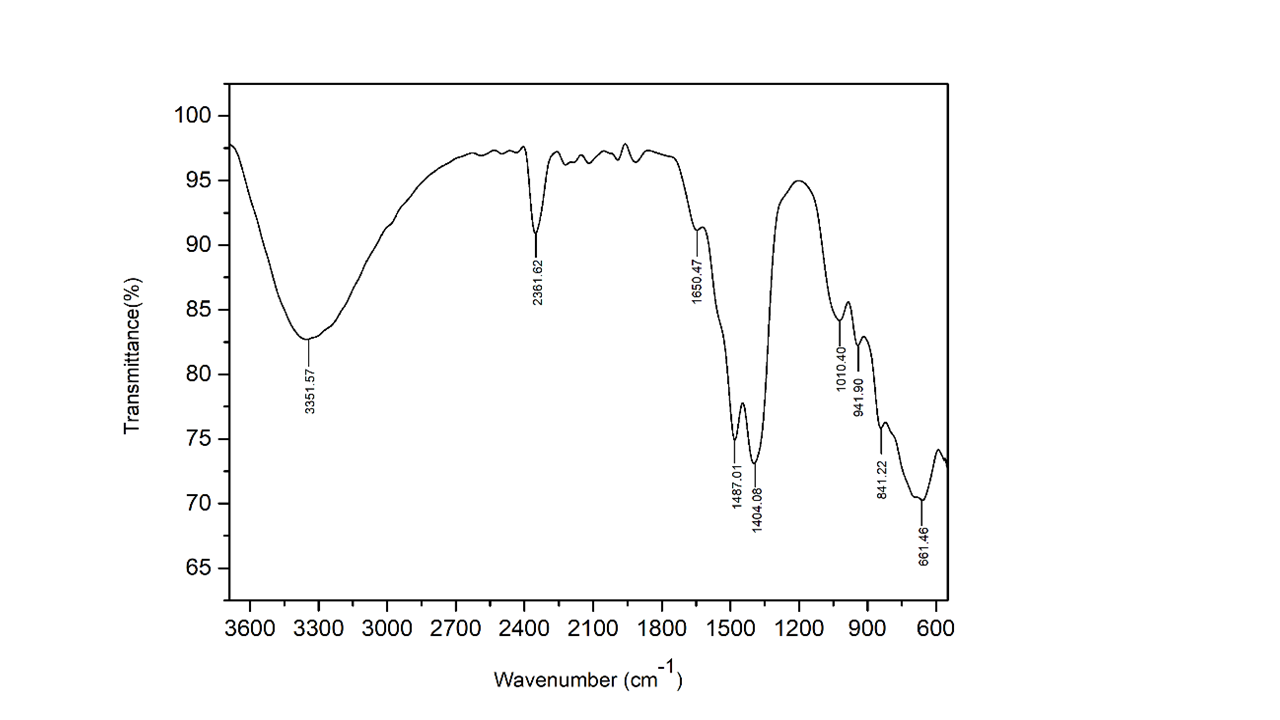


Figure S6 Infrared Fourier Transform (FT-IR) of ZnO_2_ NPs

## **9.0 Antimicrobial activities**

**Figure S7** ZnO_2_-NPs (without capping agent) against MRSA, A) agar plate well diffusion method ZOIs B) Graphical representation of ZOIs C) MIC

**Figure S8** 3% Mn ZnO_2_-NPs (without capping agent) against MRSA, A) agar plate well diffusion method ZOIs B) Graphical representation of ZOIs C) MIC

**Figure S9** 3% Mn ZnO_2_-NPs dmlt against MRSA, A) agar plate well diffusion method ZOIs B) Graphical representation of ZOIs C) MIC

**Figure S10** 5% Co-doped ZnO_2_-NPs cit against MRSA, A) agar plate well diffusion method ZOIs B) Graphical representation of ZOIs C) MIC

**Figure S11** ZnO_2_-NPs (without capping-agent) against BC, A) agar plate well diffusion method ZOIs B) Graphical representation of ZOIs C) MIC

**Figure S12** 3%Mn-doped ZnO_2_-NPs (without capping-agent) against BC, A) agar plate well diffusion method ZOIs B) Graphical representation of ZOIs C) MIC

**Figure S13** 3% Mn-doped ZnO_2_-NPs dmlt against BC, A) agar plate well diffusion method ZOIs B) Graphical representation of ZOIs C) MIC

**Figure S14** 5% Co-doped ZnO_2_-NPs cit against BC, A) agar plate well diffusion method ZOIs B) Graphical representation of ZOIs C) MIC

References

1. Jain, A., et al., *Commentary: The Materials Project: A materials genome approach to accelerating materials innovation.* APL materials, 2013. **1**(1).

2. Yabu, H., *Bottom-up approach to creating three-dimensional nanoring arrays composed of au nanoparticles.* Langmuir, 2013. **29**(4): p. 1005-1009.

3. Ali, S.S., et al., *Synthesized zinc peroxide nanoparticles (ZnO2-NPs): a novel antimicrobial, anti-elastase, anti-keratinase, and anti-inflammatory approach toward polymicrobial burn wounds.* International journal of nanomedicine, 2017: p. 6059-6073.

4. Ramírez, J.I.D.L., et al., *Synthesis and characterization of zinc peroxide nanoparticles for the photodegradation of nitrobenzene assisted by UV-light.* Catalysts, 2020. **10**(9): p. 1041.

5. Escobedo-Morales, A., et al., *Structural and vibrational properties of hydrothermally grown ZnO2 nanoparticles.* Journal of Crystal Growth, 2011. **316**(1): p. 37-41.

6. Ullah, I., et al., *Ligand-capped pristine and doped ZnO2 nanoparticles for enhanced photocatalytic methylene blue degradation: A DFT-supported study.* Ceramics International, 2025.

7. Neder, R.B. and T. Proffen, *Diffuse Scattering and Defect Structure Simulations: A cook book using the program DISCUS*. Vol. 11. 2008: OUP Oxford.

8. Eberhardt, J., et al., *AutoDock Vina 1.2. 0: New docking methods, expanded force field, and python bindings.* Journal of chemical information and modeling, 2021. **61**(8): p. 3891-3898.
